# MDP: A *Deinococcus* Mn^2+^-Decapeptide Complex Protects Mice from Ionizing Radiation

**DOI:** 10.1371/journal.pone.0160575

**Published:** 2016-08-08

**Authors:** Paridhi Gupta, Manoshi Gayen, Joan T. Smith, Elena K. Gaidamakova, Vera Y. Matrosova, Olga Grichenko, Barbara Knollmann-Ritschel, Michael J. Daly, Juliann G. Kiang, Radha K. Maheshwari

**Affiliations:** 1 Department of Pathology, Uniformed Services University of the Health Sciences (USUHS), School of Medicine, Bethesda, Maryland, United States of America; 2 Biological Sciences Group, Birla Institute of Technology and Science, Pilani, Rajasthan, India; 3 Radiation Combined Injury Program, Armed Forces Radiobiology Research Institute (AFRRI), Bethesda, Maryland, United States of America; Georgetown University, UNITED STATES

## Abstract

The radioprotective capacity of a rationally-designed Mn^2+^-decapeptide complex (MDP), based on Mn antioxidants in the bacterium *Deinococcus radiodurans*, was investigated in a mouse model of radiation injury. MDP was previously reported to be extraordinarily radioprotective of proteins in the setting of vaccine development. The peptide-component (DEHGTAVMLK) of MDP applied here was selected from a group of synthetic peptides screened *in vitro* for their ability to protect cultured human cells and purified enzymes from extreme damage caused by ionizing radiation (IR). We show that the peptides accumulated in Jurkat T-cells and protected them from 100 Gy. MDP preserved the activity of T4 DNA ligase exposed to 60,000 Gy. *In vivo*, MDP was nontoxic and protected B6D2F1/J (female) mice from acute radiation syndrome. All irradiated mice treated with MDP survived exposure to 9.5 Gy (LD_70/30_) in comparison to the untreated mice, which displayed 63% lethality after 30 days. Our results show that MDP provides early protection of white blood cells, and attenuates IR-induced damage to bone marrow and hematopoietic stem cells via G-CSF and GM-CSF modulation. Moreover, MDP mediated the immunomodulation of several cytokine concentrations in serum including G-CSF, GM-CSF, IL-3 and IL-10 during early recovery. Our results present the necessary prelude for future efforts towards clinical application of MDP as a promising IR countermeasure. Further investigation of MDP as a pre-exposure prophylactic and post-exposure therapeutic in radiotherapy and radiation emergencies is warranted.

## Introduction

Since the 1960s, the overriding goal of the field of radiobiology has been to develop medical countermeasures against ionizing radiation (IR)—for medical purposes and national defense. Currently, radiation therapy of cancers is the main contributor to radiation injury [1–4]. However, with the growing use of nuclear energy sources and the development of nuclear weapons by nations worldwide, there is a growing risk of mass exposure of the general population to IR caused by nuclear reactor accidents or acts of nuclear terrorism [5, 6]. Such incidents would demand immediate therapeutic intervention to prevent widespread morbidity and mortality caused by radiation injury [7–10]. Most radioprotection approaches tested over the last 50 years have focused on compounds which protect DNA, and compounds which modulate radiation responses [11–17]. In contrast, the current investigation is based on the discovery-driven strategy of proteome protection in IR survival [18].

Most IR toxicity models rank the genome as the most important cellular target. However, recent studies have shown that proteins, but not DNA, in the extremely radiation-resistant bacterium *Deinococcus radiodurans* are highly resistant to oxidation due to the accumulation of small-molecule manganous (Mn^2+^) antioxidants [18]. In *D*. *radiodurans*, the intracellular antioxidant complexes consist mainly of Mn^2+^, orthophosphate (Pi) and peptides, which can functionally substitute enzymes such as superoxide dismutase and catalase [19]. Mn antioxidants of *Deinococcus* spp. scavenge reactive oxygen species (ROS) generated by abiotic and biotic processes arising from exposure to gamma-radiation, ultraviolet C (UVC) radiation, and desiccation [18, 20]. In cultured mouse cells exposed to gamma-rays, protein oxidation precedes DNA damage, and is implicated as a critical and very early event in radiotoxicity [21]. Importantly, the degree to which protein oxidation is expected to influence recovery of irradiated mammalian cells is far greater than for bacteria because of the impact of genome size [18]. At doses below 30 Gy, most bacteria suffer no DNA double strand breaks (DSBs)—the most severe form of DNA damage—which would render any oxidative damage to their DSB repair enzymes inconsequential [18]. However, this is not the case for mammalian cells, where a dose as low as 1 Gy will cause ~15 DSBs, all potentially lethal events if DSB repair systems are damaged by oxidation [18]. This led to the idea of harnessing small-molecule proteome protection mechanisms of *D*. *radiodurans* as a tactic for survival in mammalian cells and animals after exposure to IR.

Earlier, Daly *et al* reported that when applied *in vitro*, naturally-occurring Mn^2+^-peptide-Pi complexes in protein-free cell extract (ultrafiltrate) of *D*. *radiodurans* protected cultured human Jurkat T-cells from 16 Gy; increased the survival of *Escherichia coli* exposed to 3 kGy; and preserved the activities of enzymes irradiated *in vitro*. [22]. *D*. *radiodurans* ultrafiltrates were found to be enriched in peptides containing aspartic acid (D), glutamic acid (E), histidine (H), glycine (G), alanine (A) and methionine (M), but not proline (P) [22]. This led to the synthesis of a rationally-designed decapeptide DEHGTAVMLK (decapeptide 1, DP1), yielding a complex named MDP, which forms spontaneously when DP1 (3 mM), MnCl_2_ (1 mM) and Pi (25 mM potassium phosphate buffer (pH 7.4)) are combined [22]. Under aqueous *in vitro* conditions, MDP protected the structure and function of irradiated proteins—purified enzymes as well as bacterial and viral proteins—exposed to massive doses of gamma-radiation [22, 23]. MDP was shown to specifically protect proteins from IR-induced damage, but did not protect DNA or RNA [23].

In this report, the peptide-components of synthetic *Deinococcus* Mn antioxidants were rationally-designed. Mn^2+^-peptide-Pi complexes were tested for their ability to protect irradiated Jurkat T-cells, and to preserve the activity of irradiated T4 DNA ligase. *In vitro*, MDP (Mn^2+^-DEHGTAVMLK-Pi) was the most radioprotective of the Mn^2+^-complexes. For the first time, MDP was tested in an animal model (B6D2F1/J (female) mice), and shown to be a safe and highly effective IR countermeasure.

## Materials and Methods

### Peptides and preparation of Mn-peptide-Pi complexes

The synthetic decapeptides DP1 (H-Asp-Glu-His-Gly-Thr-Ala-Val-Met-Leu-Lys-OH), L- and D-isomers, decapeptide DP2 (H-Pro-Gly-Pro-Gly-Pro-Gly-Pro-Gly-Pro-Gly-OH), octapeptide OP1 (H-Asp-Glu-His-Thr-Val-Met-Leu-Lys-OH), hexapeptide HP1 (H-His-Met-His-Met-His-Met-OH) and the corresponding 5FAM-labeled peptides were custom-synthesized, and the net peptide contents were determined by amino acid analysis at American Peptide Co. Inc., Sunnyvale, CA. For all peptides, the purity was ~ 95%.

Chirality of DP1-L and DP1-D isomers were confirmed by circular dichroism using a Jasco J-715 CD spectrometer (Jasco, Easton, MD). For the mouse studies, MDP was prepared as follows: DP1-L, manganese (II) chloride tetrahydrate, potassium phosphate dibasic trihydrate and potassium phosphate monobasic (Sigma-Aldrich, St. Louis, MO) were dissolved in 1 x PBS to achieve a final composition of (25 mM Pi pH 7.4, 30 mM DP1-L, 10 mM MnCl_2_). For the Jurkat T-cells and enzyme studies, see Results ‘Pre- and post-irradiation treatment with rationally-designed peptides protects human Jurkat T-cells and T4 DNA ligase’. H_2_O from Barnstead Nanopure ultrapure water purification system (Thermo Scientific, Rockford, IL) was used in all experiments.

### Jurkat T-cells

Human Jurkat T-cells (ATCC TIB-152; ATCC, Manassas, VA) were grown in RPMI 1640 medium (ATCC) with 10% fetal bovine serum (ATCC), 100 U/ml penicillin, 100 μg/ml streptomycin (Life Technology, Grand Island, NY), and maintained in a humidified 37°C incubator with continuous 5% CO_2_ supply. All peptides were dissolved in water, which was used as a vehicle for the Jurkat T-cell protection experiments. Jurkat T-cells were irradiated with ^60^Co at 12 kGy/h (Shepherd and Associates model 109–68 irradiator, San Fernando, CA) at room temperature. For cell-penetration activity, Jurkat T-cells (1.5 x 10^6^ cells/ml) were treated with the labeled peptide for 18 h, washed once with 1 x PBS, re-suspended in Hoechst 33342 (Life Technology, Eugene, OR) and mounted on a slide for microscopy. Live cell images were taken on a Leica AF-6000 microscope at 40 x magnification.

### T4 DNA ligase

T4 DNA ligase (2,000 U/μl) (New England Biolabs, Ipswich, MA) was diluted in the various reagent-mixtures to 2 U/μl for irradiation. Typically, 50 μl of the T4 DNA ligase mixtures were irradiated with ^60^Co at 12 kGy/h (Shepherd and Associates model 109–68 irradiator) aerobically on ice. Following irradiation, 5 μl of each IR-treated T4 DNA ligase sample were assayed for residual ligase activity in separate reaction mixtures (final volume, 50 μl) containing 300 ng of *Xba*I-linearized pUC19 DNA, 50 mM Tris-HCl (pH 7.5), 10 mM MgCl_2_, 10 mM dithiothreitol and 1 mM ATP (New England Biolabs). T4 DNA ligase/pUC19 DNA mixtures were incubated for 16 h at 16°C, followed by agarose (1%) gel electrophoresis.

### Animals

Female B6D2F1/J mice (12–20 week-old purchased from Jackson Laboratory, Bar Harbor, ME) were randomly assigned to experimental groups. Animal rooms were temperature-controlled at 22°C ± 4°C with 50% ± 20% relative humidity. Animals remained on a 12 h light/dark cycle and received food (Rodent diet # 8604; Harlan Teklad, Madison, WI) and water (acidified with HCl to a pH 2.5–3.0) *ad libitum*. Mice were acclimated for 1–2 weeks before any administration or exposure to IR was initiated.

#### Ethics statement

Animals were housed in micro-isolator filter-topped polycarbonate cage in a facility fully accredited by the Association for Assessment and Accreditation of Laboratory Animal Care (AAALAC)-International and treated in accordance with the guidelines from the Guide for the Care and Use of Laboratory Animals of the Institute for the Laboratory Animal Research, National Research Council. All the animal handling and euthanasia protocols were reviewed and approved by the Armed Forces Radiation Research Institute’s Institutional Animal Care and Use Committee. All efforts were made to minimize the suffering in the test animals. Animals were observed several times a day and moribund animals (and animals reaching a clinical score of ≥ 12) were immediately euthanized. Euthanasia was carried out in accordance with the recommendations and guidelines of the American Veterinary Medical Association. Briefly, animals were placed in a separate cage where carbon dioxide gas was applied until no breathing was observed, followed by a cervical dislocation as a secondary confirmatory method of euthanasia. For terminal tissue collections, animals were placed under anesthesia by isoflurane inhalation for the entire period of blood collection, immediately followed by a confirmatory cervical dislocation for euthanasia and tissue collection.

### *In vivo* study design

For all the mouse studies, MDP, which is based on the peptide DP1-L, was administered at a dose of 300 mg DP1/kg in a final volume of 200 μl. Control groups received the same volume of 1 x PBS as vehicle (since MDP is prepared using 1 x PBS).

For evaluating the toxicity of MDP complex, groups of mice (n = 6) were either administered once daily with MDP or vehicle (1 x PBS) subcutaneously (SC) for 7 days, or two oral doses (PO) on consecutive days or a combination of SC and PO administrations. Study was terminated on day 21 and blood samples were collected for further evaluation.

For the radioprotective efficacy study, groups of mice (n = 16; Vehicle+Sham (Veh+Sham), MDP+Sham, Vehicle+radiation (Veh+IR), and MDP+radiation (MDP+IR)) were given MDP or vehicle SC once daily starting from day (-1) to day 7. All animals also received MDP or vehicle orally 14 h prior to whole-body irradiation, and immediately post-irradiation. The animals were monitored at least twice daily and scored for signs of morbidity or mortality. Body weight was monitored on days 0, 1, 3, 5, 7, 14, 21 and 28. Water consumption (group mean) was measured daily with graduated bottles containing water until day 10 post-irradiation. Groups of mice were euthanized on day 1 (n = 4) and day 3 (n = 4) post-irradiation for blood and tissue collection. The study was terminated on day 30 post-irradiation, and blood and tissue samples were collected from remaining animals (n = 8) for further analysis.

#### Clinical scoring criteria

Clinical scoring for Acute Radiation Syndrome (ARS) was done from day 8 to day 30. The specific score assigned for the observed clinical features is listed in S1 table. Scores from each category were added together to obtain an overall clinical score for each animal. The higher the score, the more severely the animal was affected. Animals with an overall clinical score ≥ 12 were considered moribund and humanely euthanized immediately.

### Radiation and sham exposure *in vivo*

Thirty to forty minutes before irradiation, the mice were restrained in vertically stacked, ventilated, four compartment plexiglass boxes which provide electron equilibrium during irradiation. A single animal was placed in each compartment. Empty compartments within the boxes were filled with 3-inch long, 1-inch diameter acrylic phantoms (Precision Plastics, Inc., Beltsville, MD) to ensure uniform electron scattering. Mice were exposed to 9.5 Gy (LD_70/30_, whole-body bilateral ^60^Co gamma-radiation using Panoramic Cobalt-60 irradiator at a dose rate of 0.4 Gy/min [24]. The radiation field dosimetry was accomplished with alanine/EPR using the alanine calibration set generated from National Physical Laboratory of UK. The alanine pellets were purchased from Harwell Dosimeter (Oxfordshire, UK). The mapping provided dose rates to water in the core of the acrylic phantom in each compartment of the mouse rack on the day of irradiation. The irradiation exposure time was calculated from the mapping data. Accuracy of the actual dose delivered was verified with an ionization chamber adjacent to the mouse rack, which had been calibrated in terms of dose to soft tissue in the cores of mice. For sham exposure, animals were restrained similarly and the boxes were placed in a room next to the irradiation room for the same amount of time as the irradiated mice but did not receive any radiation dose.

### Blood toxicity profiling and complete blood count analysis

Blood samples were collected for conducting hematological and toxicity evaluation, and to isolate serum for cytokine expression and western blot analysis. Blood toxicity profile was conducted on the VITROS 350 chemistry analyzer (Ortho Clinical Diagnostics Inc., Rochester, NY) according to the manufacturer’s protocol. Differential analysis of the blood cell profile was performed on the ADIVA 2120 Hematology System (Siemens, Deerfield, IL) using the peroxidase method and the light scattering techniques recommended by the manufacturer.

### Measurement of bone marrow cell count

Bone marrow cells were collected from the femur on days 1, 3 and 30 post-irradiation. The bone marrow cells were flushed out of the femur with 3 ml of 1 x PBS twice. The cells were then centrifuged at 800 x *g* and resuspended in 10 mL 1 x PBS buffer and were counted using Countess Automated Cell Counter (Invitrogen, Grand Island, NY) (n = 4 except Veh+IR group (n = 3) on day 30). The person operating the Countess Automated Cell Counter (Life Technologies, Frederick, MD) was blinded to the study groups.

### Cytokine and chemokine measurements

Serum was separated from the whole blood of mice by centrifugation at 3000 x *g* for 10 min at 4°C and was stored at -80°C until assayed. Cytokine concentrations were measured using the BioPlex^™^ Cytokine Assay (BioRad, Hercules, CA) following the manufacturer’s directions. Data was analyzed using the LuminexH 100^™^ System (Luminex Corp, Austin, TX) and quantified using MiraiBio MasterPlexH CT and QT Software (Hitachi Software Engineering America Ltd, San Bruno, CA). Final concentrations were expressed in pg/mL unless otherwise stated (n = 4 except Veh+IR group (n = 3) on day 30).

### Histology

Sternum samples were collected on days 3 and 30 post-irradiation and were immediately fixed in 10% phosphate buffered formalin for at least 72 h. Paraffin embedded tissues were sectioned to 4 μm thickness and stained with Hematoxylin and Eosin (H&E) for histopathological analysis. Stained slides were examined under the microscope (Nikon Eclipse E400, Nikon, Instruments Inc., Melville, NY) at 4 x magnification (n = 4 on day 3 and n = 8 on day 30 except Veh+IR group (n = 3) on day 30).

### Western blot

Bone marrow cells isolated from surviving animals on day 30 were used to perform western blot analysis. Briefly, the bone marrow cells were resuspended in Hanks’ balanced salt solution (Gibco, Grand Island, NY) containing protease inhibitors and sonicated for protein isolation. Proteins in the cell lysates were separated by gel electrophoresis on 4–12% NuPAGE bis-tris precast gels (Invitrogen, Grand Island, NY) and transferred onto nitrocellulose membrane. Primary antibodies against mouse CD34 (Santa Cruz Biotechnology, Santa Cruz, CA), CD44 (Bio Legend, San Diego, CA) and IgG (Santa Cruz Biotechnology) were used for immunoblotting assays. The protein bands were quantitated using a Kodak 2200 Imager and normalized against the respective IgG levels (n = 4 except Veh+IR group (n = 3) on day 30).

### Statistical Analysis

Parametric data are expressed as the mean ± SEM (Standard Error of the Mean). The survival data were analyzed by long-rank test. For all the data analyses of blood cell counts, cytokines, western blots, and histological slides, Student’s *t*-test was used with Prism software (GraphPad, San Diego, CA) for comparison of groups, with 5% as a significant level.

## Results

### Pre- and post-irradiation treatment with rationally-designed peptides protects human Jurkat T-cells and T4 DNA ligase

**Fig 1:**
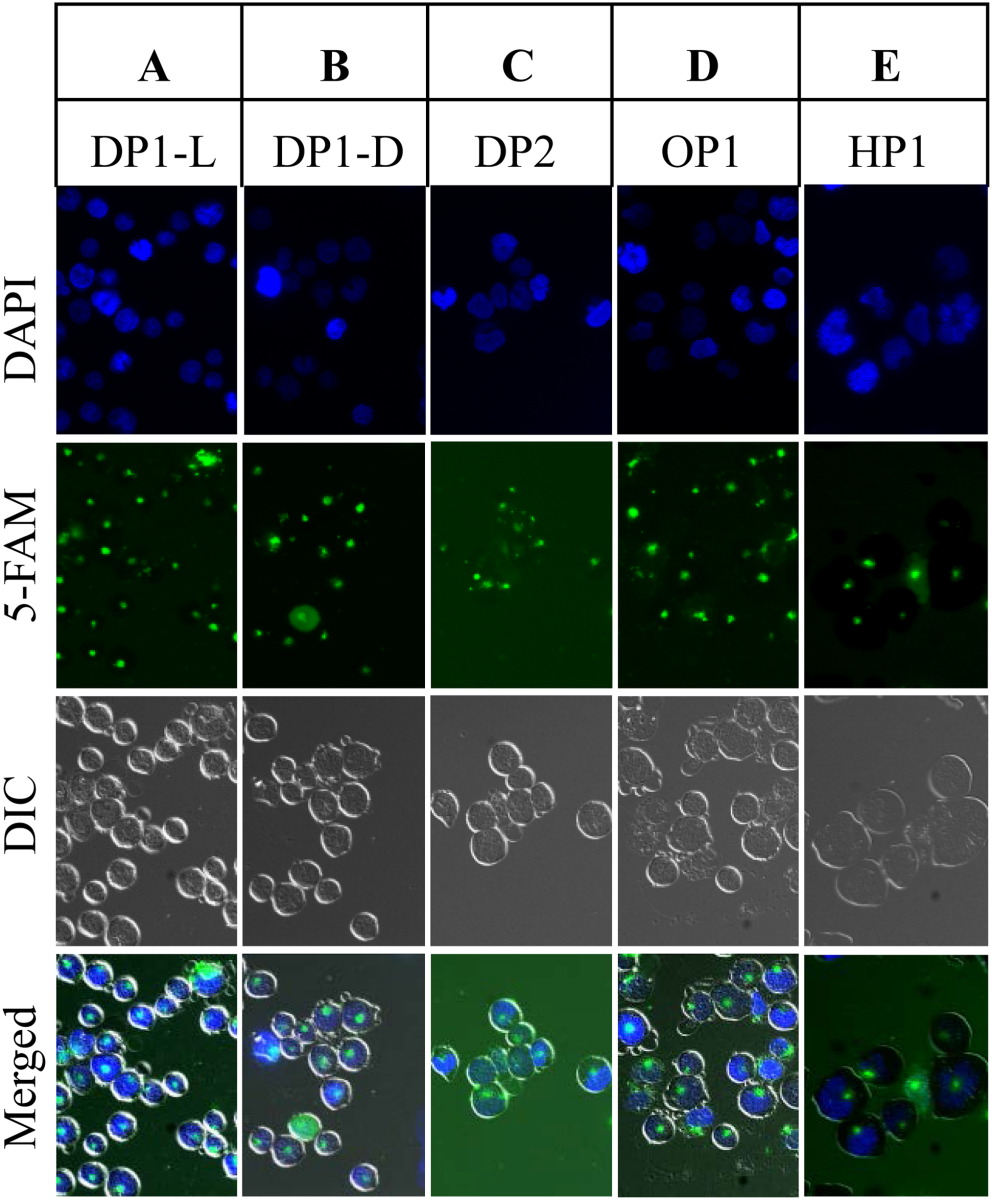
Accumulation of 5FAM-labeled peptides in Jurkat T-cells. Peptides: (A) DP1-L. (B) DP1-D. (C) DP2. (D) OP1. (E) HP1. Jurkat T-cells were incubated with the 5FAM-labeled peptides (100 μM) for 18 h. The peptides were labeled at the N-terminus with 5FAM. Note, the green signal in images corresponds to 5FAM-labeled peptides and the blue signal shows DAPI staining of the cell nucleus. The merged images show the presence of peptides inside the cells.

**Fig 2:**
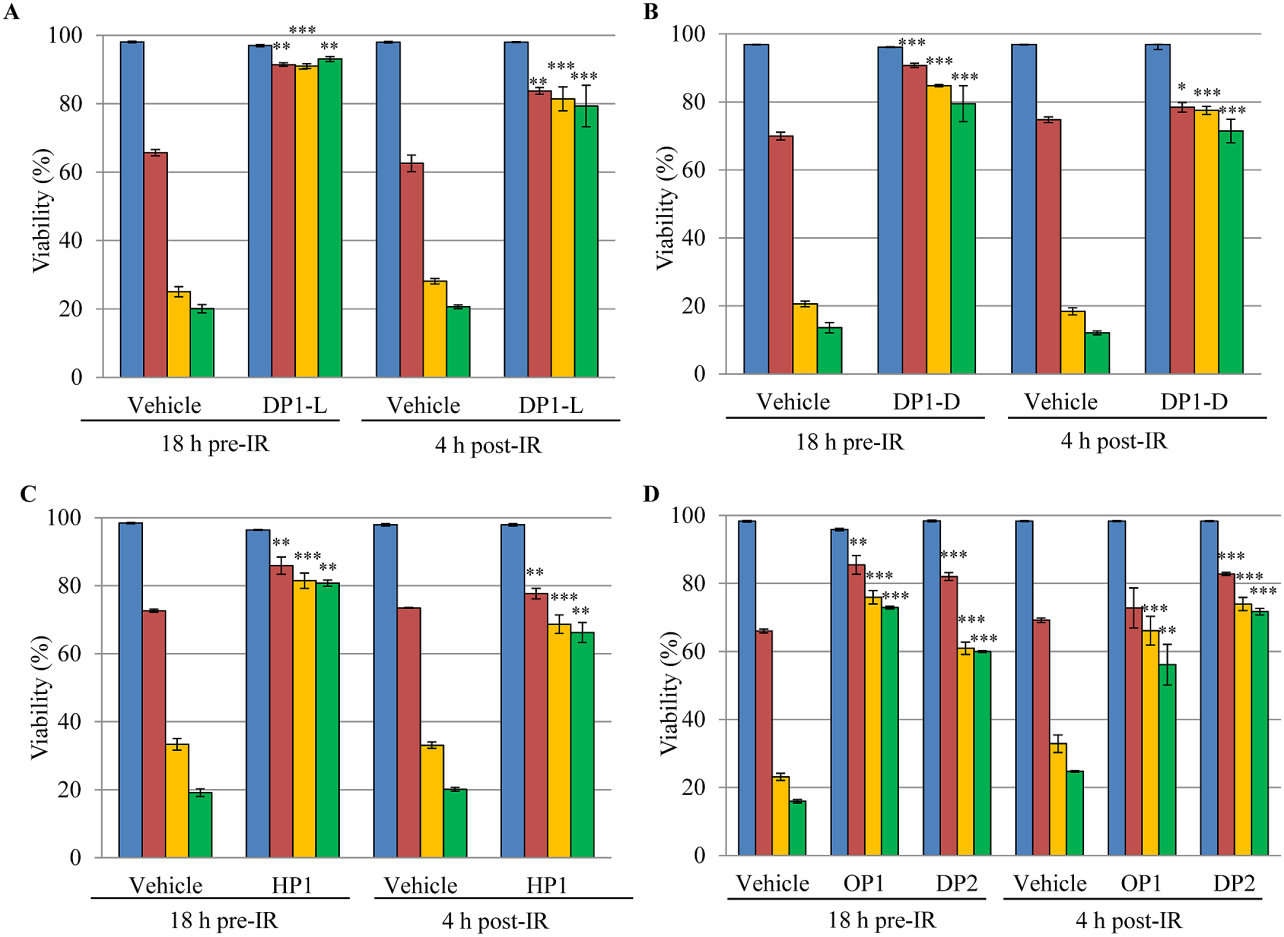
Pre- and post-irradiation treatment with rationally-designed peptides protects human Jurkat T-cells. Jurkat T-cells were treated with the indicated peptides 18 h before or 4 h after irradiation. (A) DP1-L. (B) DP1-D. (C) HP1. (D) OP1 and DP2. Final concentrations of the peptides added to RPMI were: DP1-L (3 mM); DP1-D (3 mM); HP1 (3 mM); OP1 (3.75 mM); DP2 (3 mM). Note, the final concentration of peptides in RPMI media corresponded to 30 mM of total amino acid residues, except for HP1, which was reduced to 18 mM because of toxicity of this peptide. Cell viability: blue, 0 Gy, 0 h; red, 0 Gy, 72 h; yellow, 20 Gy, 72 h; green, 100 Gy, 72 h. The experiments were carried out in triplicate with standard deviations shown. Jurkat T-cell viability was determined by SYTOX Blue staining coupled to flow cytometry at 405 nm. Asterisks indicate p-value compared to vehicle group (*p < 0.05, **p < 0.01, ***p < 0.001).

Cell-penetration by the rationally-designed peptides DP1 (L- and D-isomers), HP1, OP1, and DP2 was examined by the addition of fluorescently-labeled peptides (5FAM) to human Jurkat T-cells, followed by tracking the labeled peptides in the cells by fluorescent microscopy. All five labeled peptides penetrated and were stably accumulated in the cells after 18 h (Fig 1, merged images). We then tested the relative radioprotective efficacy of the corresponding unlabeled peptides (DP1-L, DP1-D, DP2, OP1 and HP1) on the viability of irradiated Jurkat T-cells cultured *in vitro* (Fig 2 and S1 Fig). It should be noted, the peptides alone were added to complete RPMI medium, which contains sufficient Mn^2+^, Pi and bicarbonate to form complexes [22, 23]. The peptides were added to the cell cultures either 18 h before or 4 h after irradiation at doses of 0 Gy, 20 Gy and 100 Gy. The viability of Jurkat T-cells was assayed by SYTOX Blue (S1 Fig), which readily penetrates mammalian cells with compromised plasma membranes; such cells are dead. For cells which are metabolically-active and have intact membranes, SYTOX Blue will not cross membranes; such cells remain metabolically active, but may not be replication-proficient (*i*.*e*., for cells that have accumulated an insurmountable number of IR-induced DSBs).

The results show that DP1-L and DP1-D conferred the greatest levels of radioprotection on human Jurkat T-cells, among the different peptides tested (Fig 2). In general, D-forms of organic molecules are not readily metabolized by cells [25]. This would be expected to increase the intracellular and extracellular half-life of DP1-D over DP1-L. However, the IR responses of irradiated Jurkat T-cells treated with DP1-L or DP1-D (0.1–3.0 mM) were essentially the same (S2 Fig). The order of radioprotection of Jurkat T-cells by the five peptides was as follows: DP1-L > DP1-D > OP1 ≈ HP1 > DP2.

We previously showed that near-100% of Jurkat T-cells treated *ex vivo* prior to irradiation with *D*. *radiodurans* ultrafiltrate survived 16 Gy, but with no significant protection observed at 20 Gy or greater [22]. Here we show that the levels of radioprotection conferred on Jurkat T-cells by five rationally-designed peptides (Fig 2 and S1 Fig) far exceed the levels bestowed by *D*. *radiodurans* ultrafiltrate [22]. Following exposure of Jurkat T-cells in RPMI medium to 100 Gy, cell viability was reduced to 10–20% after 72 h of recovery (Fig 2 and S1 Fig). Remarkably, the addition of 3 mM DP1 (-L or -D) as a radioprotector to RPMI 18 h prior to irradiation increased the viability of Jurkat T-cells exposed to 100 Gy from ~20% to ~90% (Fig 2A and 2B and S1 Fig). Moreover, the viability of non-irradiated Jurkat T-cells incubated in RPMI for 72 h was consistently greater in the presence of DP1 (-L or -D) than without DP1 (Fig 2A and 2B, and S1A and S1B Fig). Importantly, Jurkat T-cells that were treated 4 h post-irradiation with 3 mM DP1, but not before irradiation, displayed similarly high levels of viability (~80%) following 100 Gy (Fig 2A and 2B and S1A and S1B Fig).

**Fig 3:**
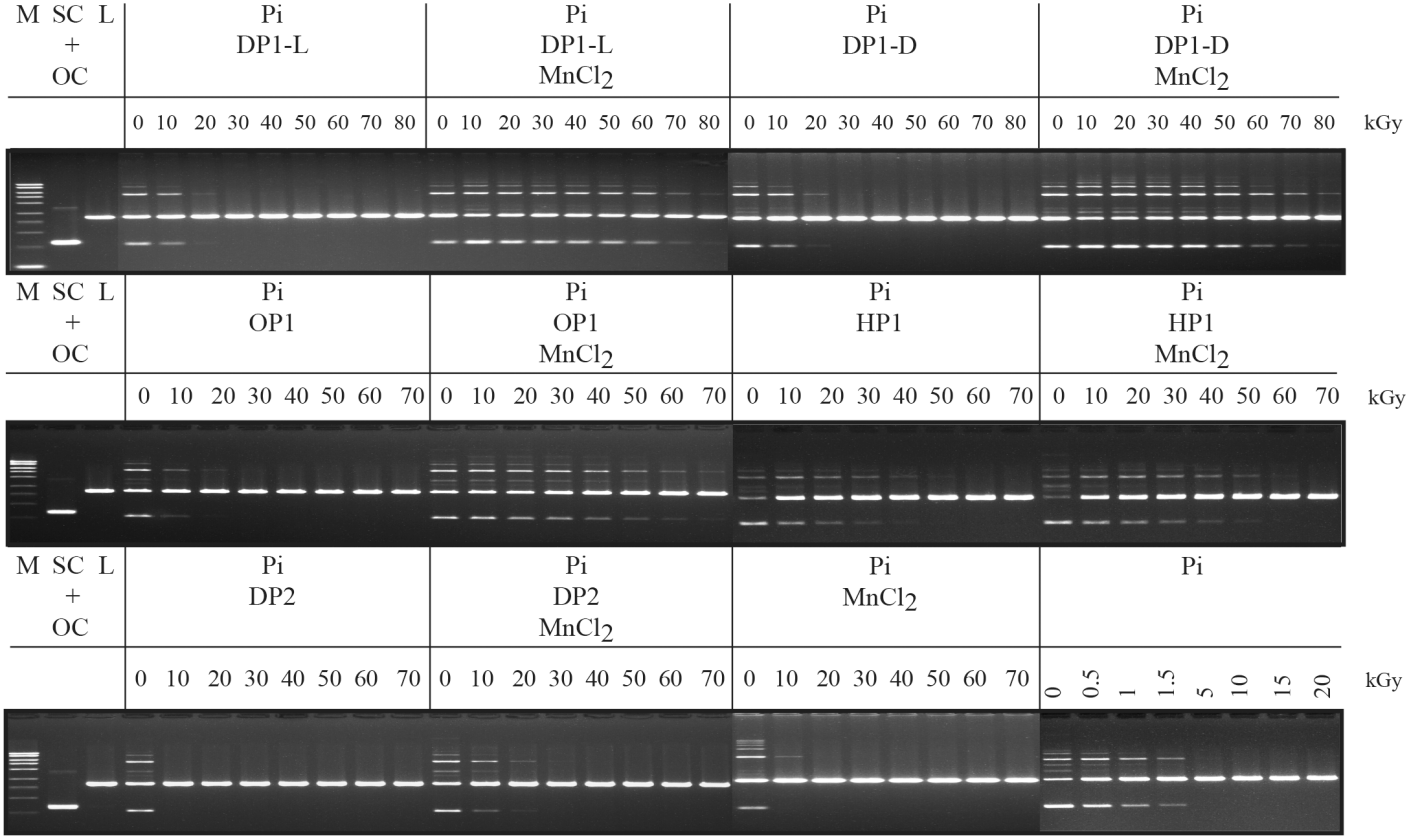
Radioprotection of T4 DNA ligase by rationally-designed peptides. T4 DNA ligase was irradiated in the indicated mixtures and then tested for residual activity by incubation with linearized (L) pUC19 DNA (2.686 kbp). Formation of supercoiled (SC) and open-circular (OC) DNA forms were determined by agarose gel electrophoresis. Peptides were added to 25 mM Pi with or without 1 mM MnCl_2_ to the following concentrations: DP1-L, 3 mM; DP1-D, 3 mM; DP2, 3 mM; OP1, 3.75 mM; HP1, 5 mM. Note, the final concentration of peptides in the reaction mixtures corresponded to 30 mM of total amino acid residues. Other abbreviations: Pi, potassium phosphate buffer (pH 7.4); M, DNA size standards (Mass Ruler DNA Ladder mix; Fermentas, Glen Burnie, MD).

Peptides tested for their ability to protect Jurkat T-cells from IR were also evaluated for their ability to preserve the activity of T4 DNA ligase, which served as a proxy DNA repair enzyme *in vitro* (previous enzyme-targets were metabolic enzymes and restriction endonucleases [22]). In the absence of IR, T4 DNA ligase joins ends of pUC19 plasmid DNA linearized with *Xba*I, yielding open-circular and supercoiled products that are readily distinguished from the linear form by gel electrophoresis (Fig 3). When prepared in 25 mM Pi buffer pH 7.4 in the absence of Mn^2+^ and peptides, the dose of gamma-radiation required to extinguish the activity of T4 DNA ligase was approximately 1.5 kGy (Fig 3). T4 DNA ligase irradiated in 25 mM Pi with 1 mM MnCl_2_ showed some activity after 10 kGy. When peptides were added, protection increased dramatically. DP1-L or DP1-D mixed with MnCl_2_ in Pi buffer showed by far the highest level of radioprotection of T4 DNA ligase at doses up to 60 kGy (Fig 3). For irradiated Jurkat T-cells, DP1-L was the most radioprotective (Fig 2). Thus, we chose DP1-L for our radioprotection study using a mouse model of radiation injury.

### MDP is safe in mice

**Fig 4:**
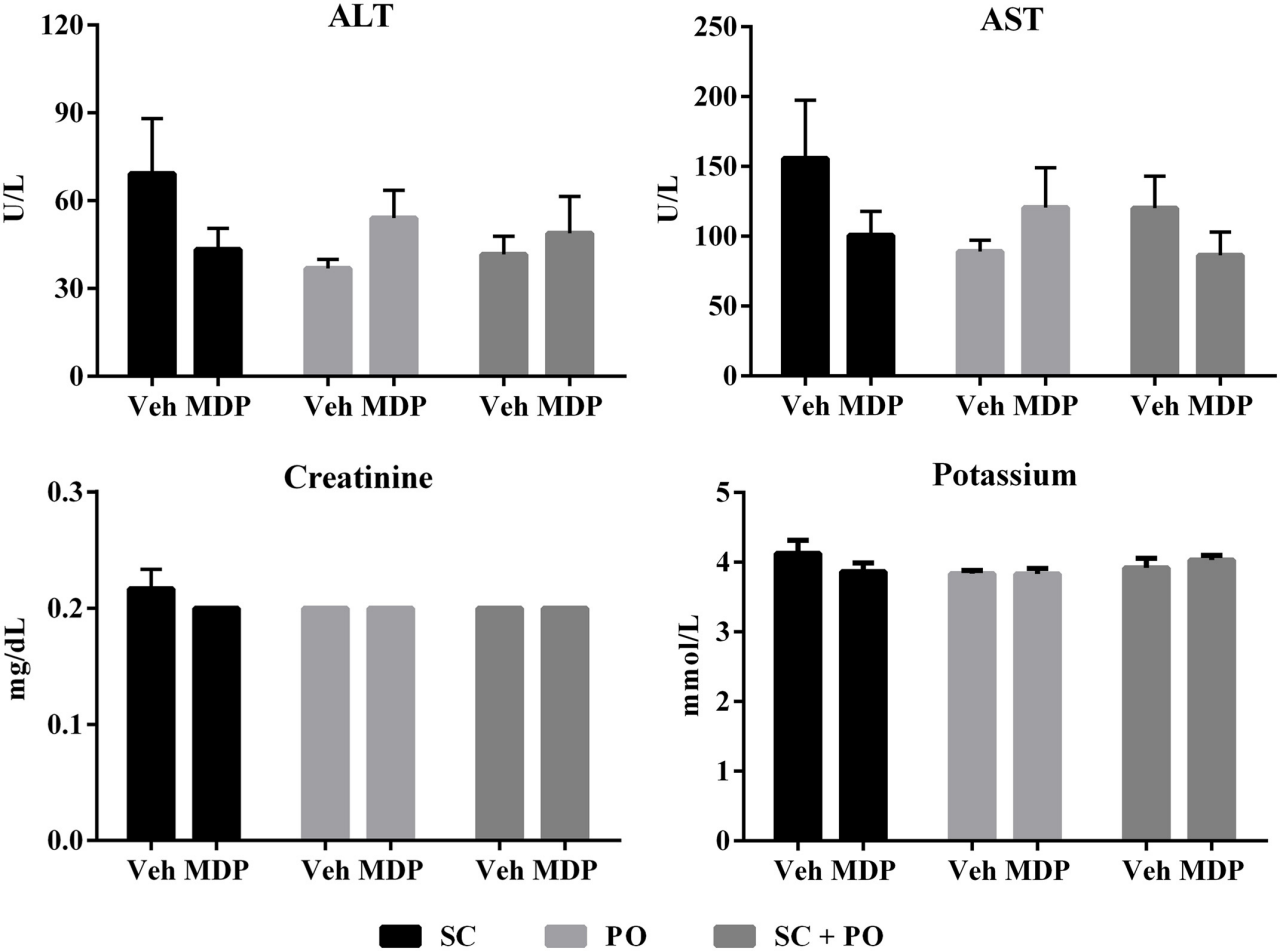
Expression levels of injury markers after MDP administration. Modulation of the various markers of injury (A) ALT, (B) AST, (C) Creatinine and (D) Potassium were evaluated in the blood after MDP or vehicle administration. *p < 0.05 vs. Veh via same route. Abbreviations: Veh, vehicle; SC, subcutaneous; PO, by mouth.

Based on the rankings of *in vitro* radioprotection assays (Figs 2 and 3), MDP (MnCl_2_-(DP1-L)-Pi) was evaluated for its ability to protect mice from IR-induced morbidity and mortality. In order to determine any toxic effect of MDP administration *in vivo*, B6D2F1/J female mice were administered with MDP at a dose of 300 mg DP1/kg either SC for seven consecutive days or PO for two days, or a combination of both SC and PO administrations. All of the animals survived until the end of the study. No significant difference was observed in weight gain or water intake over a 14-day observation period (S3 Fig). Expression of hepatic injury markers (ALT, alkaline phosphatase, AST, albumin, bilirubin and protein), renal injury markers (chloride, calcium, CO_2_, glucose, potassium, phosphorus, sodium, uric acid and urea nitrogen) and additional toxicity markers (HDLC, LDH and creatine kinase) was evaluated in serum samples collected from the animals at the end of the study. The expression levels of the injury markers showed no adverse effect of treatment on mice with MDP (Fig 4 and S4 Fig). Therefore, the results indicate that MDP is nontoxic *in vivo*.

### MDP confers radioprotection in mice

**Fig 5:**
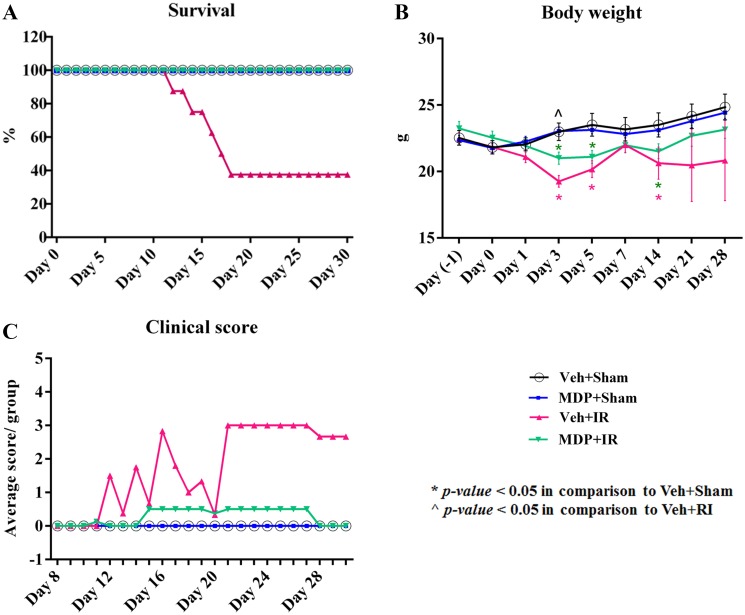
MDP confers radioprotection *in vivo* and results in improved clinical scores in animals post-irradiation. (A) 100% 30-day survival in MDP-treated irradiated mice. (B) Reduced body weight loss in MDP-treated irradiated mice. (C) Lower average clinical scores in MDP-treated irradiated mice. All the animals were monitored daily for any clinical signs of morbidity or mortality associated with IR exposure, and scored accordingly. Details of the scoring criteria are described in S1 Table. Animals with an overall clinical score ≥ 12.0 were considered moribund and immediately humanely euthanized. IR exposure dose was 9.5 Gy. *p < 0.05 vs. Veh+Sham; ^*p* < 0.05 vs. Veh+IR. Abbreviation: Veh, vehicle.

The *in vivo* radioprotective efficacy of pre- and post-exposure treatment with MDP was tested in B6D2F1/J female mice against whole body radiation exposure to 9.5 Gy (LD_70/30_). Our results show that a combination of pre- and post-irradiation administration of MDP at 300 mg DP1/kg conferred 100% survival against IR-induced mortality (Fig 5A). In contrast, 63% of the vehicle-treated irradiated animals succumbed to IR-induced injury between days 12 and 18 post-irradiation. There was no mortality in the sham irradiated groups (MDP treated or vehicle treated non-irradiated animals).

Ionizing radiation induced significant loss of body weight from day 3 post-irradiation in the vehicle-treated animals. However, MDP administration significantly prevented the IR-induced weight loss on day 3 post-irradiation resulting in faster recovery of these animals (21.0 g vs. 19.2 g, *p* < 0.05) (Fig 5B). Total body weight for MDP-treated irradiated animals was similar to that of the sham irradiated groups by day 21, whereas the survivors of vehicle-treated irradiated animals had persistent lower body weight extending through day 30 post-irradiation. MDP treatment had no effect on water consumption (S5 Fig). Clinical scoring for ARS was done daily from day 8 to day 30 based on the evaluation of the overall appearance, respiratory rate, general behavior, provoked behavior and weight loss in the animals to evaluate possible effects of MDP treatment on IR-induced morbidity in the surviving animals. Daily clinical scoring of the animals indicated that MDP administration delayed the onset of ARS and facilitated recovery, whereas the survivors of the vehicle-treated group maintained high clinical scores due to IR-induced morbidity until the end of the study (Fig 5C). Therefore, MDP not only provided protection against IR exposure, but also significantly reduced morbidity in the surviving animals.

### MDP ameliorates IR-induced leukocytopenia, erythrocytopenia and splenomegaly

**Fig 6:**
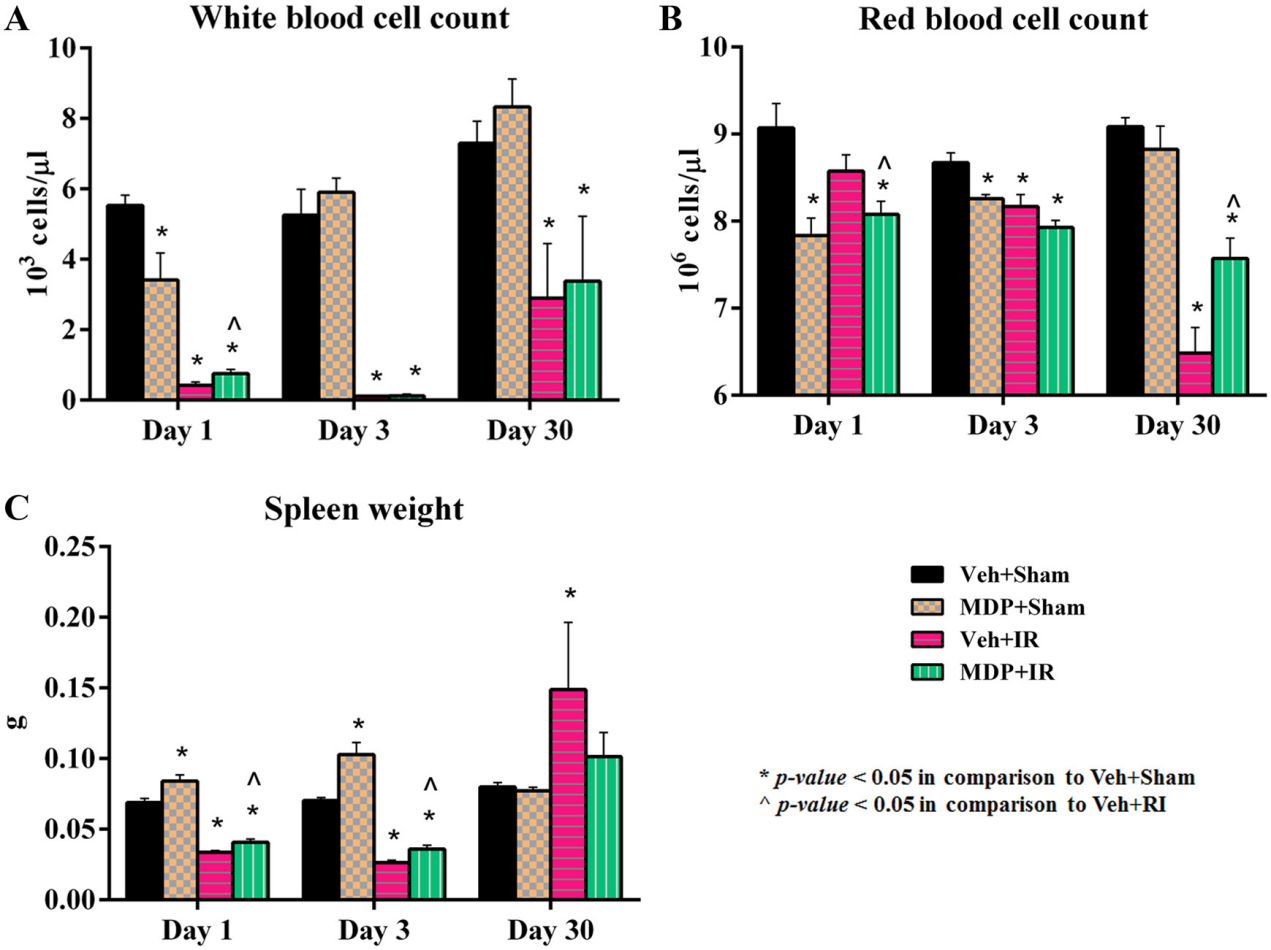
MDP ameliorates IR-induced leukocytopenia, erythrocytopenia and splenomegaly. (A) Attenuation of IR-induced WBC loss in MDP-treated irradiated mice. (B) Attenuation of IR-induced RBC loss in MDP-treated irradiated mice. (C) Inhibition of splenomegaly by MDP treatment. IR exposure dose was 9.5 Gy. *p < 0.05 vs. Veh+Sham; ^*p* < 0.05 vs. Veh+IR. Abbreviation: Veh, vehicle.

During ARS, different subsets of circulating peripheral blood cells (*i*.*e*., total white blood cells (WBCs), lymphocytes, monocytes, neutrophils, eosinophils, basophils, and platelets) are known to be depleted. To test whether or not MDP-treatment ameliorated the leukopenia, total blood cell counts were measured at various time-points post-irradiation to estimate the number of different blood cell types remaining in each group. MDP treatment significantly improved the IR-induced loss of total WBCs immediately after radiation exposure on day 1 (Fig 6A). Exposure to IR also induces anemia via decline in red blood cell (RBC) count, hemoglobin level and hematocrit levels. There was significant elevation in the RBC count as well as increased levels of hemoglobin and hematocrit in MDP-administered animals by day 30 post-irradiation in comparison to the untreated animals (Fig 6B and S6 Fig).

MDP administration prevented IR-induced splenomegaly, *i*.*e*., enlargement of the spleen in the irradiated animals measured at day 30 post-exposure unlike vehicle-treated animals (Fig 6C), which correlated with the recovery of circulating RBCs at this time point. Therefore, MDP administration ameliorated IR-induced leukocytopenia initially post-irradiation and erythrocytopenia and splenomegaly later during recovery from radiation injury. Improvement in all these vital parameters by MDP administration may be responsible for recovery of the animals, thereby preventing death.

### MDP administration prevents bone marrow cell depletion, adipogenesis and reduction in CD34 expression

**Fig 7:**
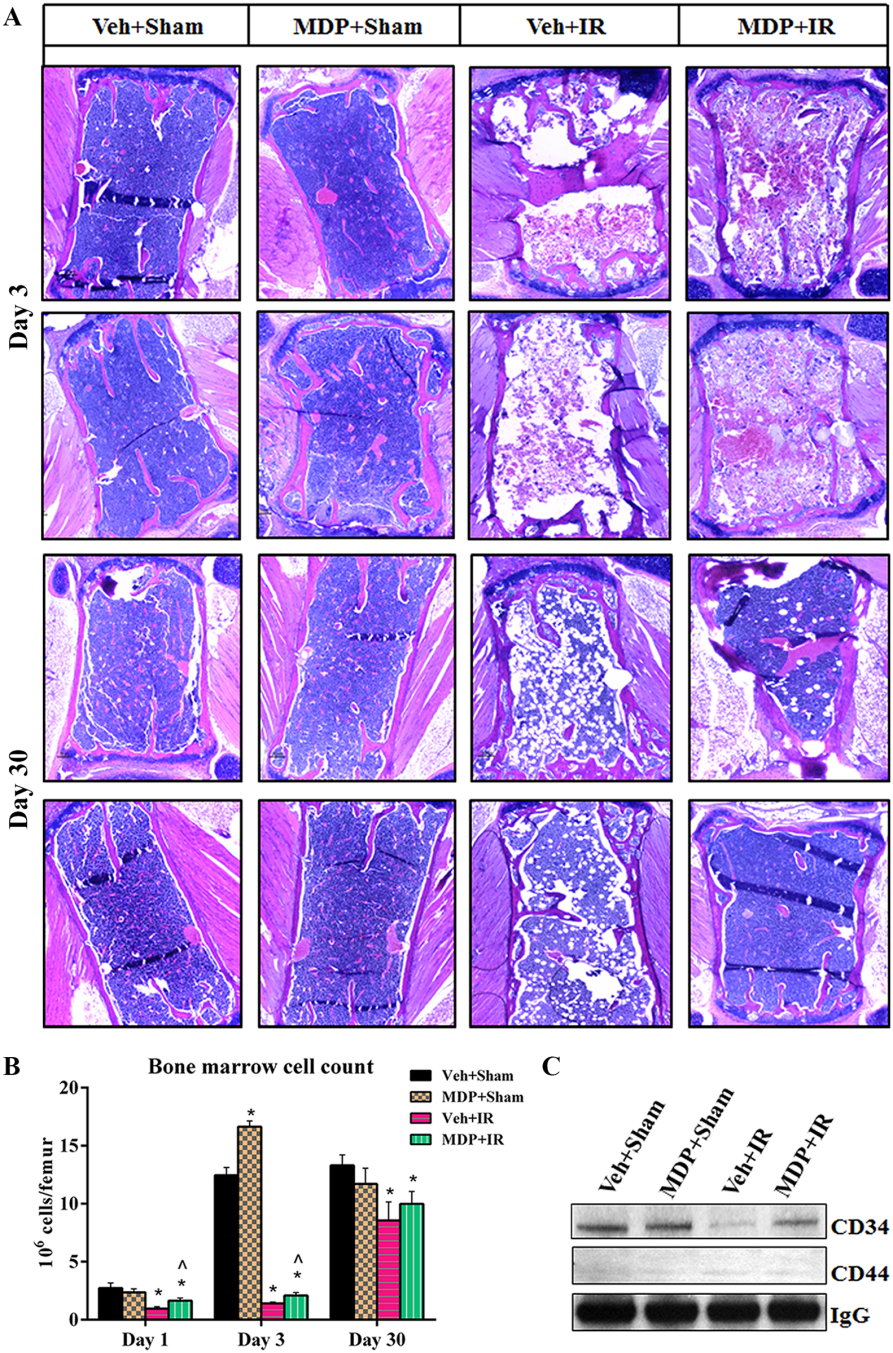
MDP administration prevents bone marrow cell depletion, adipogenesis and reduction in CD34 expression. (A) MDP administration prevented the IR-induced bone marrow cell loss and adipogenesis. Representative figures are shown from multiple biological replicates. (B) Bone marrow cell counts in mice treated with either MDP or Veh after sham or IR exposure. (C) Alterations in the expression levels of CD34 and CD44 at day 30 post-irradiation. Results are representative of similar observations from multiple replicates. IR exposure dose was 9.5 Gy. *p < 0.05 vs. Veh+Sham; ^*p* < 0.05 vs. Veh+IR. Abbreviation: Veh, vehicle.

Ionizing radiation induces loss of bone marrow cells. Bone marrow cell counts and evaluation of histopathological changes by H&E staining in vehicle-treated mice showed that IR caused loss of bone marrow cells and led to the formation of numerous fat cells by day 30 (Fig 7A). In comparison, MDP administration significantly ameliorated IR-induced loss of bone marrow cells in mice on day 1 and day 3 post-irradiation, and resulted in higher bone marrow cell counts and significantly decreased adipogenesis on day 30 (Fig 7A and 7B). In order to identify the sub-population of bone marrow cells protected by MDP, the expression levels of the mesenchymal stem cell surface markers CD34 (hematopoietic lineage marker) and CD44 (matrix receptor) were analyzed by western blot. MDP selectively resulted in ~3 times greater expression of CD34 compared to vehicle administration, but no significant change was observed in CD44 expression levels, which indicates that hematopoietic progenitor stem cells expressing CD34 in the bone marrow were protected (Fig 7C and S7 Fig). Substantially reduced numbers of IR-induced fat cells observed in H&E-stained bone marrow sections of MDP-treated animals, together with elevated levels of CD34, are indicators of the protection of bone marrow cells from radiation injury by MDP. Taken together, these results support that MDP may work as a “hematopoietic stem cell protector”.

### MDP administration modulates cytokine response *in vivo*

**Fig 8:**
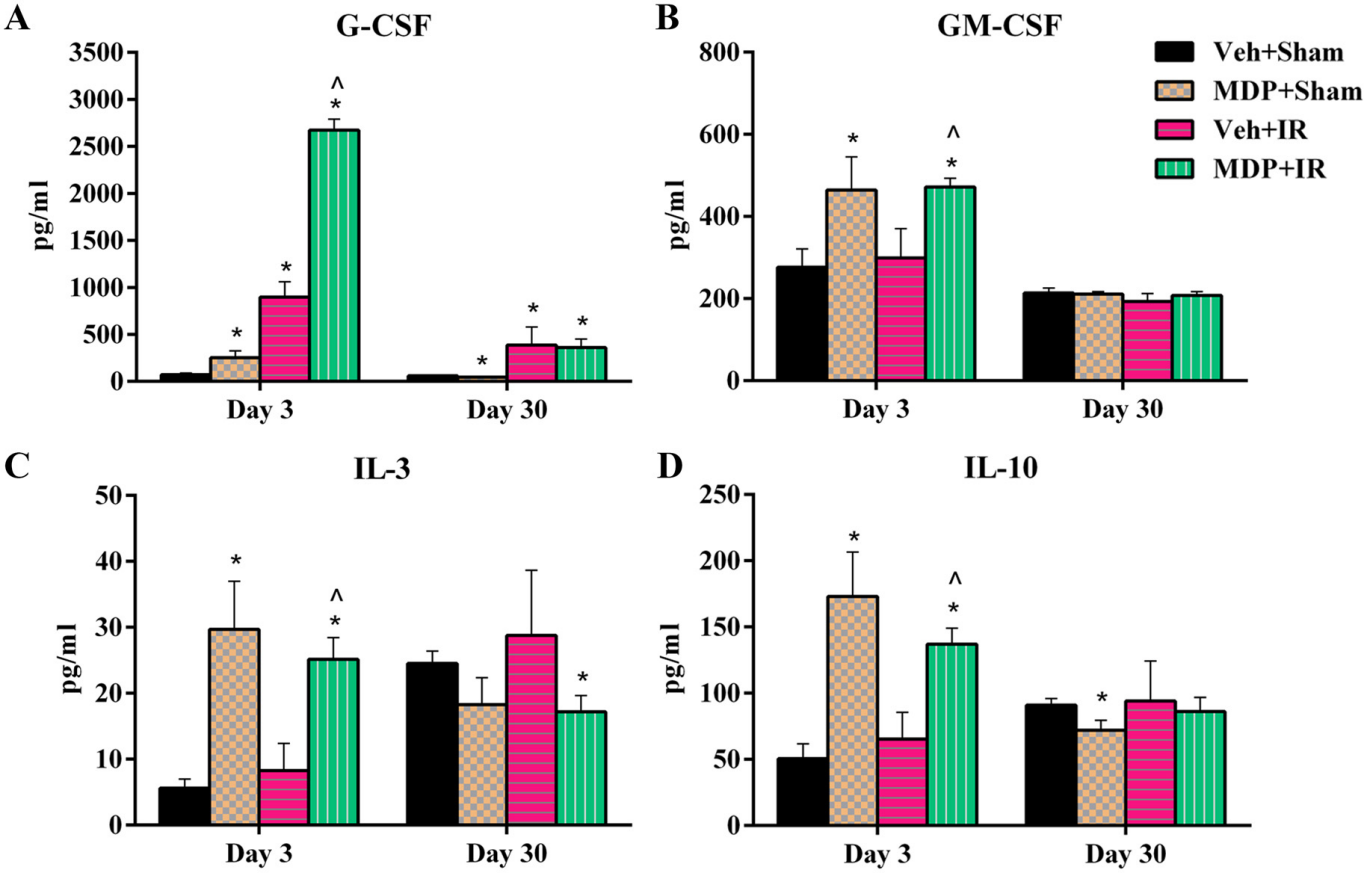
MDP administration modulates cytokine response *in vivo*. Modulation of cytokines in the serum of irradiated mice on days 3 and 30 was evaluated by Luminex multiplex. (A) G-CSF level. (B) GM-CSF level. (C) IL-3 level. (D) IL-10 levels. IR exposure dose was 9.5 Gy. *p < 0.05 vs. Veh+Sham; ^*p* < 0.05 vs. Veh+IR. Abbreviation: Veh, vehicle.

Several cytokines including IL-2, -3, -4, and -10, granulocyte colony stimulating factor (G-CSF), and granulocyte macrophage colony stimulating factor (GM-CSF) were found to be significantly up-regulated upon MDP administration on day 3 in all mice, while most of the cytokines returned to their basal levels by day 30 in surviving animals (Fig 8 and S8 Fig). The cytokines, IL-3, IL-10, G-CSF and GM-CSF, were the most up-regulated by MDP administration in the irradiated mice. Interestingly, MDP treatment alone without irradiation also enhanced expression of these serum cytokines on day 3, however they came back to basal level by day 30.

## Discussion

After decades of research, very few treatment options exist to combat the effects of whole-body irradiation [11, 17]. With the growing risk of mass exposure to life-threatening doses of IR, there is an urgent need to develop effective, nontoxic medical countermeasures [6, 8, 26]. A variety of synthetic compounds, as well as those derived from natural sources, have been tested for their radioprotective potential *in vitro* as well as *in vivo*. Several recently published review articles provide more detailed lists of such compounds [12–15]. At present, Androstenediol, OrbeShield^™^, BIO 300, CBLB502, HemaMax^™^ and Ex-RAD are some potential radioprotectors which are under Investigational New Drug (IND) status, but require further investigation to be approved by the US Food and Drug Administration (FDA). Although a number of potential drug candidates have been shown to be radioprotective in animal models, most cause significant side effects and are still under pre-clinical or clinical evaluation [2, 11, 24, 27–30]. For example, Amifostine is FDA-approved to treat xerostomia (dryness of mouth) after head and neck radiotherapy, but it commonly is accompanied with side effects such as nausea, vomiting, chills, fever, dizziness, hypotension and skin rashes [27, 31–34]. NEUPOGEN^®^ (filgrastim) and NEULASTA^®^ (pegfilgrastim), recently approved by the FDA to treat acute hematopoietic syndrome and neutropenia, respectively, also cause side effects such as bone pain and pain in extremities coupled with other risks such as fatal splenic rupture, acute respiratory distress syndrome, fatal sickle cell crisis, serious allergic reactions, and glomerulonephritis [35, 36]. Therefore, the need to develop safer and more effective treatments for IR injury persists.

The remarkable capacity of synthetic *Deinococcus* Mn antioxidants to scavenge IR-induced ROS served as the basis for this pilot study, which initially ranked rationally-designed Mn^2+^-peptide-Pi complexes for their ability to preserve the viability of irradiated Jurkat T-cells (Fig 2 and S1 Fig), and also for their capacity to protect irradiated T4 DNA ligase (Fig 3). This led to the selection of MDP for *in vivo* testing in a mouse model of radiation injury.

For all cell-types, chromosomal DNA is an indispensable molecule whose integrity must be conserved following exposure to IR to ensure survival. Thus, any process that inhibits the activity of DNA repair–for example, by mutation of repair genes or by oxidative inactivation of repair enzymes–will limit a cell’s ability to recover from genome damage [18]. A previous study reported that amino acid sequence-variants of MDP are extremely radioprotective of the metabolic enzyme glutamine synthetase *in vitro* [22, 37]. Consistently, we show that MDP is extremely radioprotective of purified T4 DNA ligase (Fig 3), a DNA repair enzyme. Before moving to an animal model, we tested the rationally-designed peptides for their ability to protect irradiated Jurkat T-cells *in vitro*. Among the peptides which could penetrate and accumulate in cultured Jurkat T-cells (Fig 1), DP1 was the most radioprotective: DP1 (H-Asp-Glu-His-Gly-Thr-Ala-Val-Met-Leu-Lys-OH) L- and D-isomers > OP1 (H-Asp-Glu-His-Thr-Val-Met-Leu-Lys-OH) = HP1 (H-His-Met-His-Met-His-Met-OH) > DP2 (H-Pro-Gly-Pro-Gly-Pro-Gly-Pro-Gly-Pro-Gly-OH) (Fig 2). However, Mn-peptide-Pi complexes do not significantly protect DNA from IR damage [22, 23]. Exposure to 100 Gy causes approximately 1,500 DSBs/haploid genome in Jurkat T-cell (~0.005 DSB/Gy/Mbp), a yield that far exceeds the DSB repair capacities of even the most radiation-resistant prokaryotes (*e*.*g*., *D*. *radiodurans*) and eukaryotes (*e*.*g*., *Ustilago maydis*) [18]. Remarkably, the addition of 3 mM DP1 18 h prior to irradiation or 4 h post-irradiation increased the viability of Jurkat T-cells from 20% to nearly 90% and 80%, respectively, when exposed to 100 Gy. Moreover, the presence of DP1 enhanced the viability of non-irradiated Jurkat T-cells.

The consequences of IR exposure of an individual are very complex due to different factors: absorbed radiation dose in tissues, the diverse array of cellular responses to absorbed doses in tissue micromasses, and the downstream effects of the various individual cellular responses on the whole tissue and organs [5, 17]. The toxicity assay of MDP *in vivo* exhibited no significant difference in the renal, hepatic and other injury marker panels (Fig 4 and S4 Fig). Interestingly, administration of MDP was found to be associated with beneficial effects such as reduction in the levels in blood of CO_2_, blood urea nitrogen, blood bilirubin, blood LDH, blood creatine kinase and blood uric acid levels, and increasing blood calcium levels. A combination of pre- and post-exposure therapy with the MDP (300 mg DP1/kg) until 7 days post-irradiation protected the mice from whole body exposure to 9.5 Gy (LD_70/30_), conferring 100% survival in MDP-treated animals versus 37% survival in the vehicle-treated group. MDP not only conferred complete protection against radiation injury but also prevented the morbidity caused by IR exposure. The irradiated animals treated with MDP were much healthier and did not suffer for a long time in contrast to the survivors of IR exposure that were not treated with MDP. These results warrant further studies on MDP, including interrogating the concentration-dependent effects of MDP on IR-survival for animals at LD_30/30_ and LD_50/30_ doses, administered before and after irradiation, or separately before as a prophylactic or after irradiation as a therapeutic drug.

Acute radiation syndrome is associated with body weight loss, vomiting, diarrhea, leukocytopenia, thrombocytopenia, erythropenia, splenomegaly, and gastrointestinal injury [30, 38–40]. The bone marrow is the most radiation-sensitive organ in mammalian hematopoietic systems due to the rapid turnover of the cells. Exposure to IR typically results in depletion of bone marrow cells and hematopoietic progenitor cell reservoirs, causing a sharp decline in the circulating blood cell count very early during the post-irradiation period [39, 41]. We show that MDP administration significantly protected the bone marrow against radiation injury and reduced IR-induced WBC depletion on day 1 post-irradiation. Thus, decreased hematopoietic stem cell injury by MDP may have facilitated the restoration of WBCs post-irradiation. Compared with the non-treated group, MDP treatment of the mice preserved the RBC count together with hemoglobin and hematocrit levels in radiation injury animals at late time-points. This supports a possible role of MDP in late mitigation of IR-induced anemia. Exposure to IR also causes splenomegaly (*i*.*e*., enlargement of the spleen) due to accumulation of both damaged and normal erythrocytes, together with excessive storage of platelets targeted for destruction in the spleen, which in turn reduces the number of healthy RBCs circulating in the blood stream [24, 29, 42]. The restoration of normal spleen weight in the irradiated group by MDP, versus splenomegaly in the untreated radiation group on day 30 post-irradiation, is consistent with the recovery of circulating RBC levels in MDP-treated irradiated mice. MDP also facilitated recovery from IR-induced thrombocytopenia, although there was no effect on the restoration of neutrophils in comparison to the untreated controls.

Ionizing radiation-induced loss of erythroid and myeloid cells and megakaryocytes leads to the formation of fat vacuoles in bone marrow [43]. MDP treatment significantly ameliorated IR-induced loss of bone marrow cellularity, and distinctly reduced the IR-induced adipogenesis in MDP-treated animals. Furthermore, MDP treatment was observed to accelerate the recovery of bone marrow cellularity post-irradiation. Sepsis and weakened immunity due to loss of hematopoietic progenitor cells contributes to IR-induced lethality [44, 45]. Modulation of hematopoietic progenitor cells in mammals is crucial for survival and recovery from IR injury during the initial days post-irradiation [30, 39, 40]. Expression of CD34 indicates the presence of healthy hematopoietic, mesenchymal and other bone marrow cells [46]. The higher bone marrow cell count post-irradiation in MDP-treated animals is consistent with elevated CD34 levels and reduced adipogenesis. The results indicate that MDP may work as a “hematopoietic cell protector” via modulation of CD34, though the exact mechanism remains to be elucidated.

G-CSF and GM-CSF are known to initiate proliferation and differentiation of myeloid progenitor cells and help in mobilizing hematopoietic stem cells from the bone marrow into the bloodstream [24, 38, 47]. IL-3 is known to regulate the differentiation of multipotent hematopoietic stem cells into myeloid progenitor cells, similar to the effects of GM-CSF [48, 49]. Administration of G-CSF and GM-CSF has yielded promising results in restoring circulating blood cells in various animal models, as well as in human subjects exposed to IR, and thus recently attained FDA-approval for treatment of ARS [24, 35, 50, 51]. Generally, radioprotectors which increase G-CSF levels elicit better protection against IR-induced bone marrow damage than those which do not [24]. The increases we observed in CD34 levels, the enhanced protection of WBCs at early time points, and the improvement of bone marrow cellularity all correlate with G-CSF-up regulation in MDP-treated mice. The exceptional “hematopoietic” radioprotection by MDP in irradiated mice reinforces the idea that G-CSF and GM-CSF are important modulators in mammalian IR resistance responses.

In conclusion, this is the first study to show that a rationally-designed Mn^2+^-decapeptide-Pi complex, MDP, which is based on Mn antioxidants found in the extremely radiation-resistant bacterium *D*. *radiodurans*, is nontoxic and prevents IR-induced injury in mice. This study reveals various potential mechanisms through which MDP may confer radioprotection *in vivo*. Certainly, additional studies on MDP are warranted to explore its use as a radioprotector or mitigator in other animal models. Given the urgency to develop safe and efficient radiation countermeasures, these results give realistic hope that *Deinococcus* Mn-peptide antioxidants will confer IR resistance on humans, and that antioxidant treatments based on MDP, which specifically protect proteins from IR-induced ROS, represent a new approach to preventing ARS. At this point, there are few alternatives to the presented perspectives on responding rapidly to radiation emergencies.

## Supporting Information

S1 Fig Pre- and post-irradiation treatment with peptides protects human Jurkat T-cells from IR. Jurkat T-cells were treated with the indicated peptides 18 h before or 4 h after irradiation. (A) DP1-L. (B) DP1-D. (C) DP2. (D) OP1. (E) HP1. Final concentrations of the peptides added to RPMI were: DP1-L (3 mM); DP1-D (3 mM); HP1 (3 mM); OP1 (3.75 mM); DP2 (3 mM). The final concentration of peptides in RPMI media corresponded to 30 mM of total amino acid residues, except for HP1, which was reduced to 18 mM because of toxicity. The SYTOX Blue staining profiles from which data in the Fig 2 were constructed.
(DOCX)

S2 Fig Concentration-dependent effects of DP1-L and DP1-D on the viability of Jurkat T-cells post-IR. The viability of Jurkat T-cells was determined by SYTOX Blue staining coupled to flow cytometery at 405 nm. Jurkat T-cells were treated 18 h before IR. IR doses: 20 Gy (A) and 100 Gy (B). The experiments were carried out in triplicate with standard deviations shown.
(DOCX)

S3 Fig MDP is safe *in vivo*. (A) Comparison of the body weight and (B) Evaluation of the difference in water consumption by different groups of MDP- or Vehicle-treated mice. *p < 0.05 vs. Veh via same route. Veh, vehicle; SC, subcutaneous; PO, by mouth.
(DOCX)

S4 Fig Expression levels of injury markers after MDP administration. Modulation of the various markers of injury albumin, alkaline phosphatase, bilirubin, calcium, CO2, creatine kinase, HDLC, glucose, LDH, phosphorus, protein, sodium, urea nitrogen and uric acid was evaluated in the blood after MDP or vehicle administration. *p < 0.05 vs. Veh via same route. Veh, vehicle; SC, subcutaneous; PO, by mouth.
(DOCX)

S5 Fig MDP has no effect on water consumption post-irradiation. Daily water consumption by each animal was measured for the first 10 days post-irradiation. IR exposure dose was 9.5 Gy. *p < 0.05 vs. Veh+Sham; ^*p* < 0.05 vs. Veh+IR. Abbreviation: Veh, vehicle.
(DOCX)

S6 Fig Complete blood cell count. Differential cell counting was assayed with blood of the different treatment groups post-irradiation. (A) Neutrophil count, (B) Lymphocyte count, (C) Basophil count, (D) Monocyte count, (E) Eosinophil count, (F) Platelet count, (G) Hemoglobin and (H) Hematocrit levels in blood post-irradiation. IR exposure dose was 9.5 Gy. *p < 0.05 vs. Veh+Sham; ^*p* < 0.05 vs. Veh+IR. Abbreviation: Veh, vehicle.
(DOCX)

S7 Fig Quantitation of CD34 and CD44 expression levels in bone marrow cells. (A) Correlation in the CD34/IgG ratios of the CD34 western blot of bone marrow lysates. (B) Correlation in the CD44/IgG ratios of the CD44 western blot of bone marrow lysates. IR exposure dose was 9.5 Gy. Abbreviation: Veh, vehicle.
(DOCX)

S8 Fig Modulation of cytokines and chemokines after MDP administration. Modulation of cytokine and chemokine expression was assayed in serum on days 3 and 30 post-irradiation by Luminex multiplex array. The panel displays the modulation of (A) IL-1α. (B) IL-1ß. (C) IL-2. (D) IL-4. (E) IL-5. (F) IL-6. (G) IL-8 (KC). (H) IL-9. (I) IL-12p40. (J) IL-12p70. (K) IL-13. (L) IL-17a. (M) IFN-γ. (N) TNF-α. (O) MCP-1. (P) Eotaxin. (Q) Rantes. (R) MIP-1α. (S) MIP-1β. IR exposure dose was 9.5 Gy. *p < 0.05 vs. Veh+Sham; ^*p* < 0.05 vs. Veh+IR. Abbreviation: Veh, vehicle.
(DOCX)

S1 Table Clinical scoring criteria. Clinical scoring for Acute Radiation Syndrome (ARS) was done from day 8 to day 30. The table summarizes the clinical scoring criteria and specific score assigned for various categories evaluated. Scores from each category were added together to obtain an overall clinical score for each animal. Animals with an overall clinical score ≥ 12 were considered moribund and humanely euthanized immediately.
(DOCX)
