# Supplementary figures and images for: MDP: A Deinococcus Mn2+-Decapeptide Complex Protects Mice from Ionizing Radiation
Source: PLoS One. 2016 Aug 8;11(8):e0160575. doi: 10.1371/journal.pone.0160575 (PMC4976947; doi:10.1371/journal.pone.0160575)

**S1 Fig:**

**
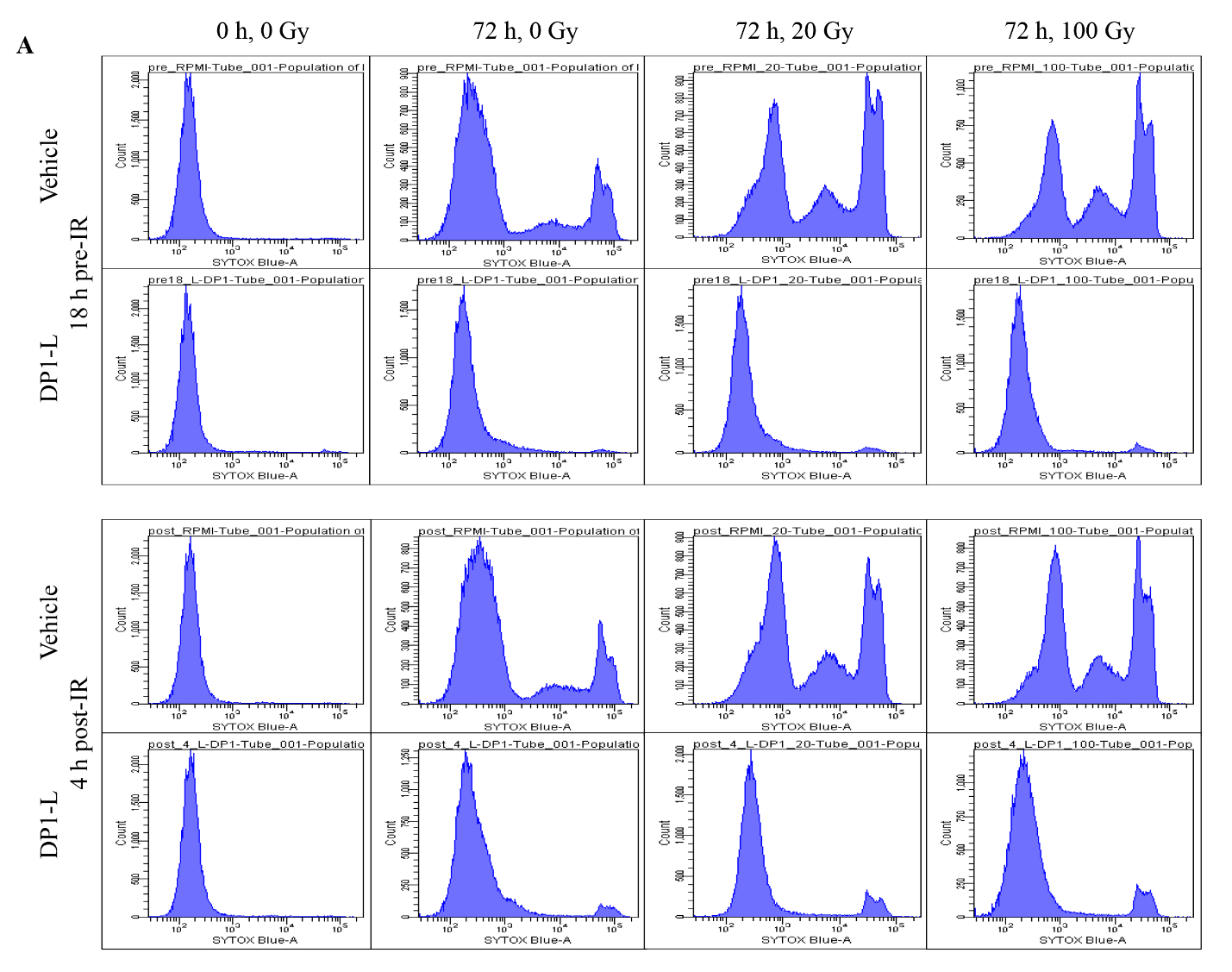
**

**
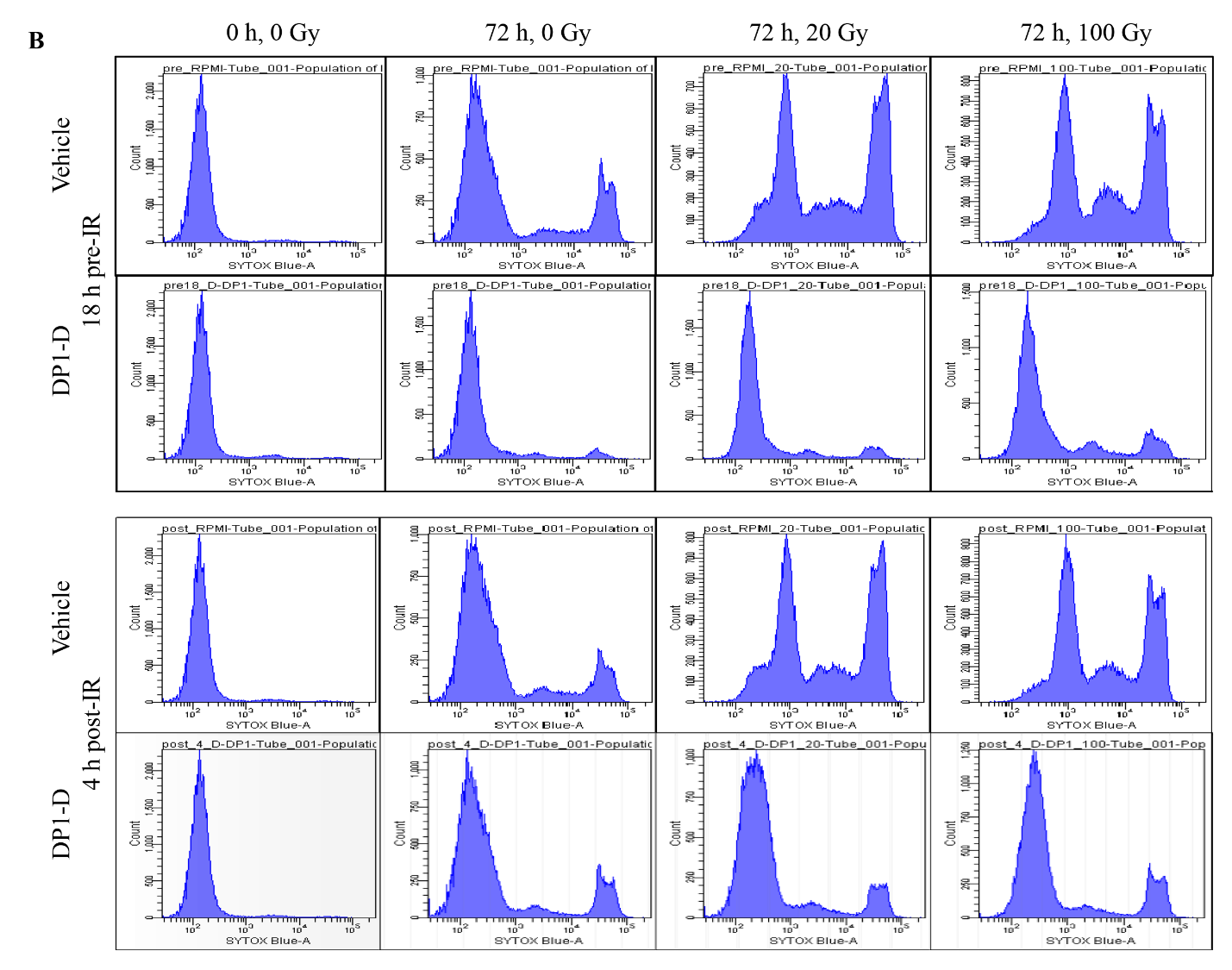
**

**
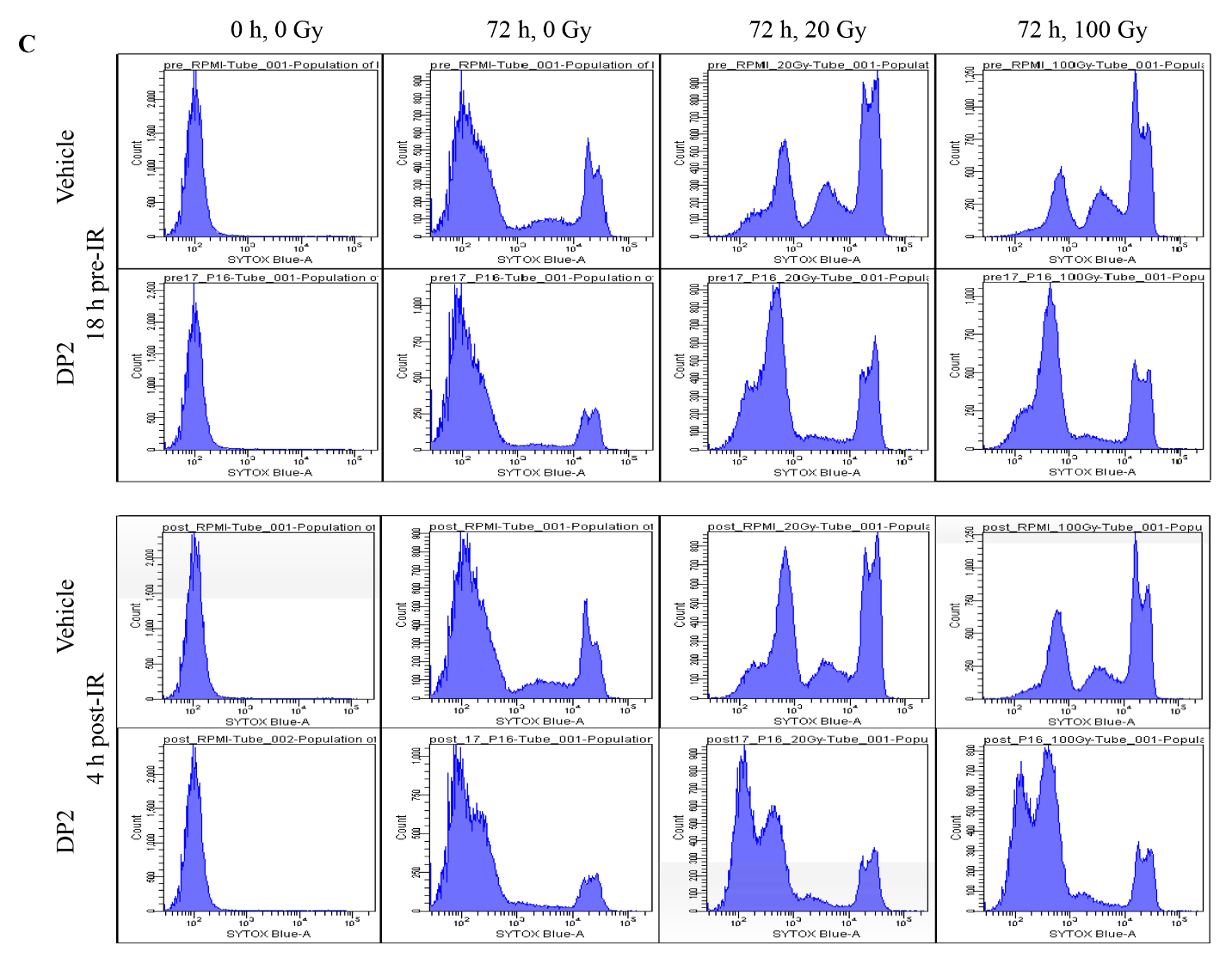
**

**
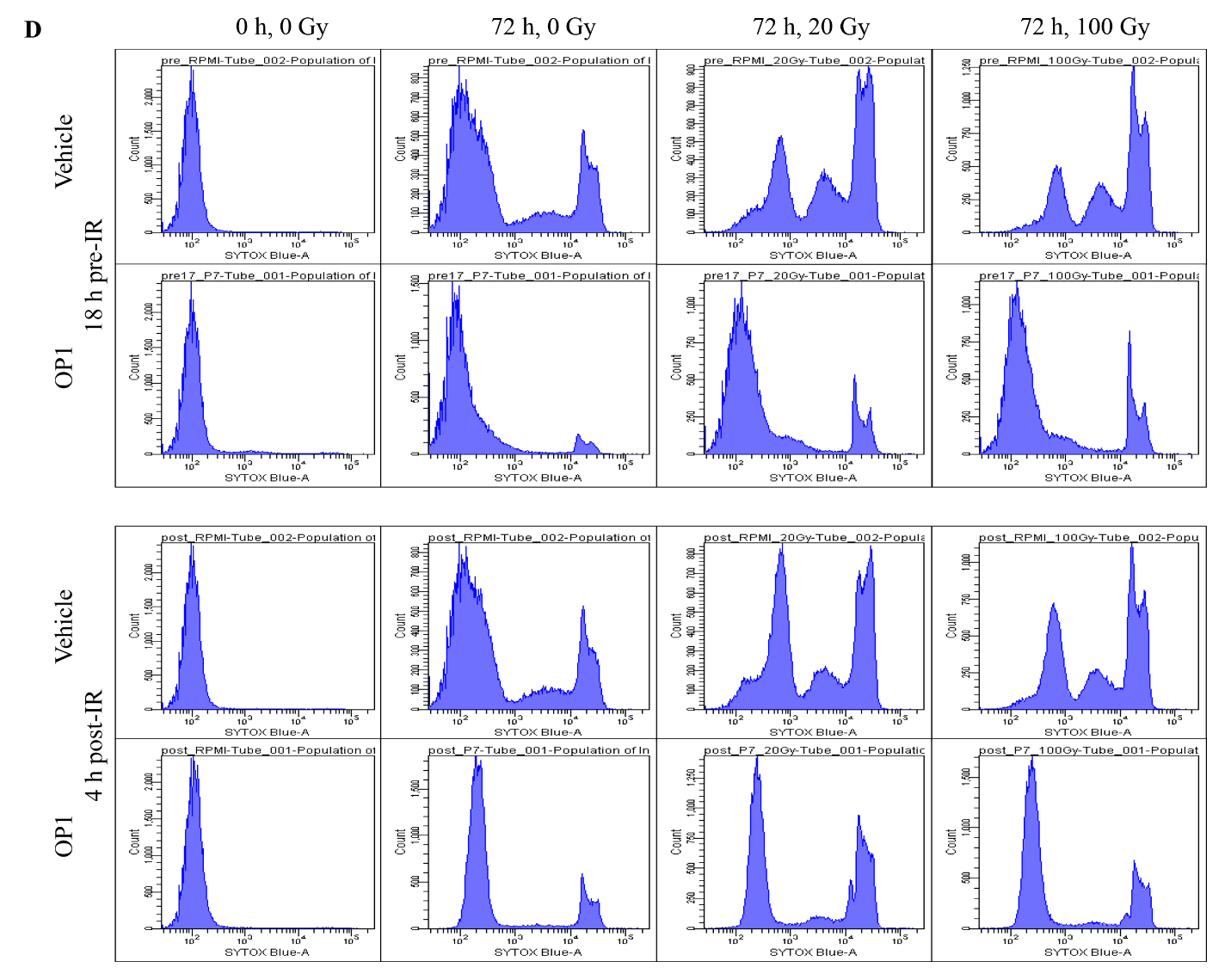
**

**
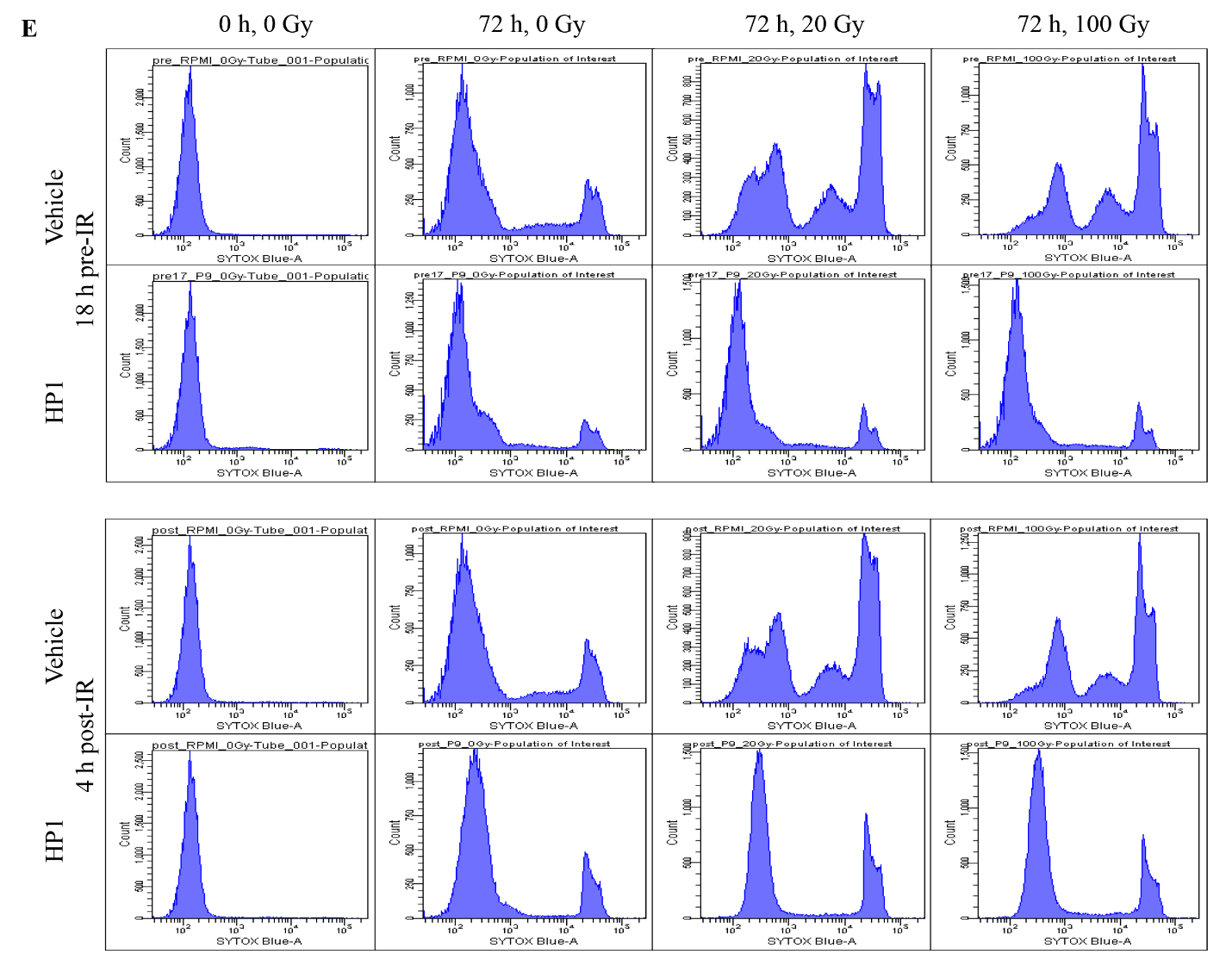
**

Supplement: S1 Fig — Jurkat T-cells were treated with the indicated peptides 18 h before or 4 h after irradiation. (A) DP1-L. (B) DP1-D. (C) DP2. (D) OP1. (E) HP1. Final concentrations of the peptides added to RPMI were: DP1-L (3 mM); DP1-D (3 mM); HP1 (3 mM); OP1 (3.75 mM); DP2 (3 mM). The final concentration of peptides in RPMI media corresponded to 30 mM of total amino acid residues, except for HP1, which was reduced to 18 mM because of toxicity. The SYTOX Blue staining profiles from which data in the Fig 2 were constructed. (DOCX) [file pone.0160575.s001.docx]

**S2 Fig:**

**
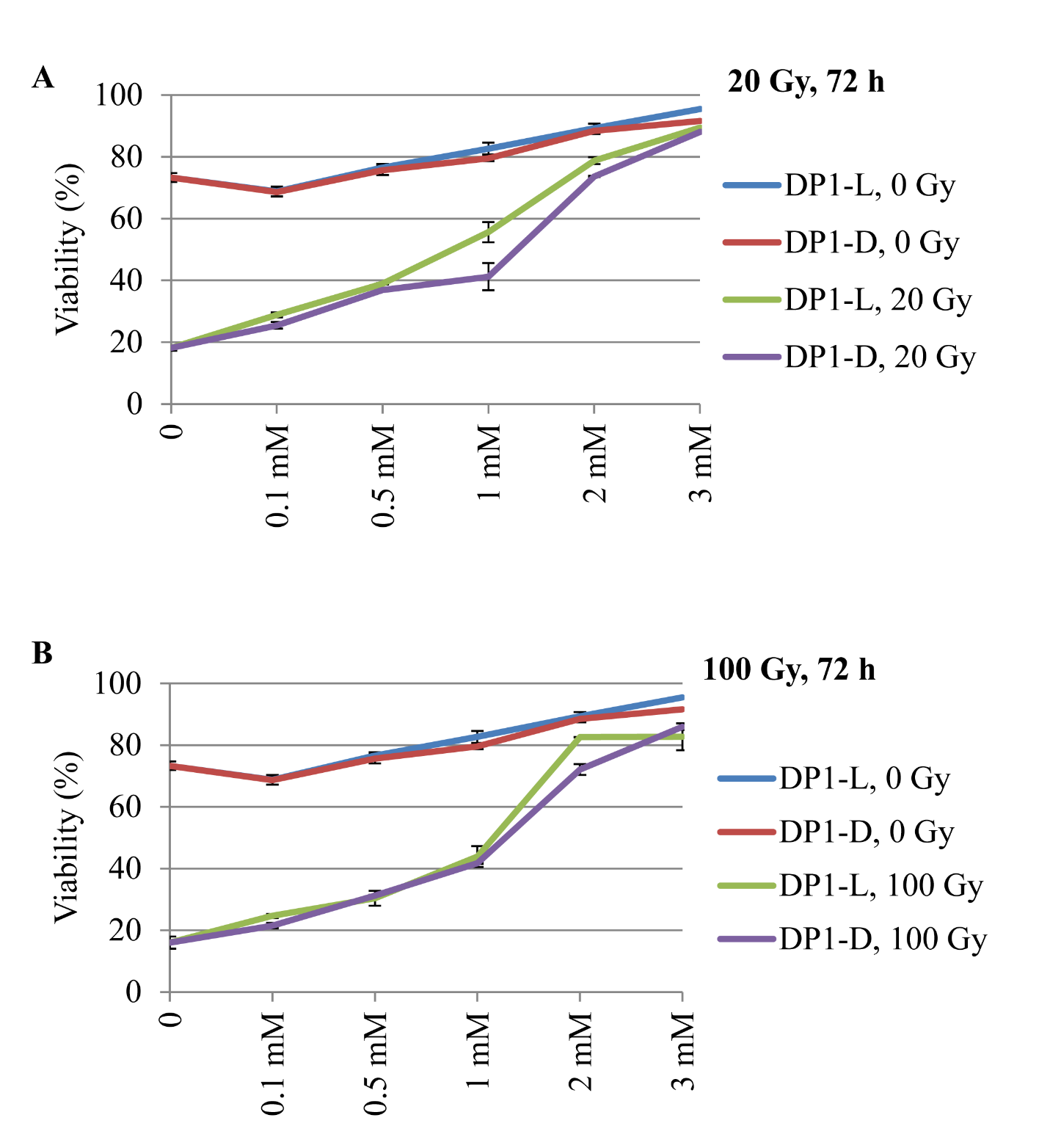
**

Supplement: S2 Fig — The viability of Jurkat T-cells was determined by SYTOX Blue staining coupled to flow cytometery at 405 nm. Jurkat T-cells were treated 18 h before IR. IR doses: 20 Gy (A) and 100 Gy (B). The experiments were carried out in triplicate with standard deviations shown. (DOCX) [file pone.0160575.s002.docx]

**S3 Fig:**

**
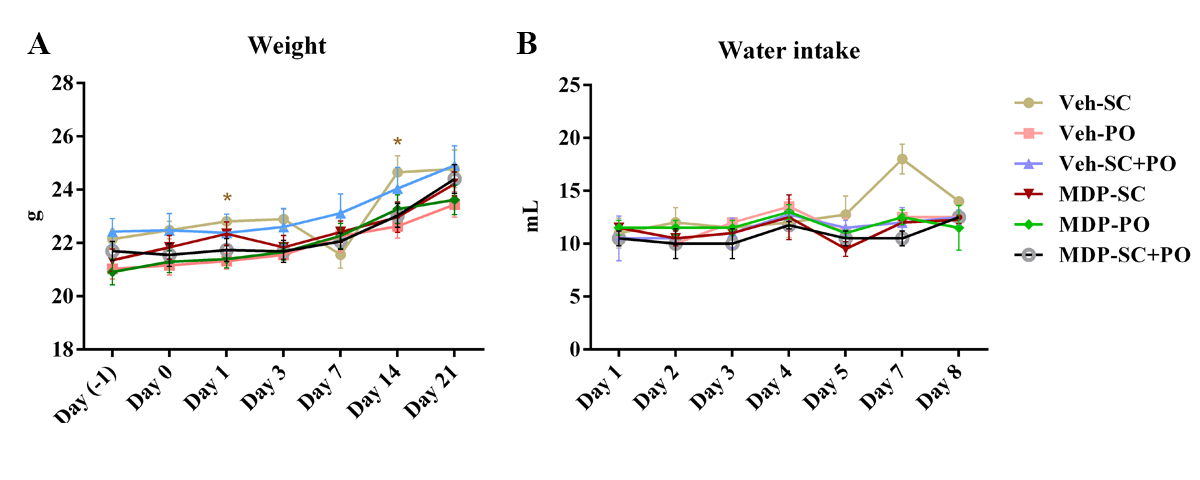
**

Supplement: S3 Fig — (A) Comparison of the body weight and (B) Evaluation of the difference in water consumption by different groups of MDP- or Vehicle-treated mice. *p < 0.05 vs. Veh via same route. Veh, vehicle; SC, subcutaneous; PO, by mouth. (DOCX) [file pone.0160575.s003.docx]

**S4 Fig:**

**
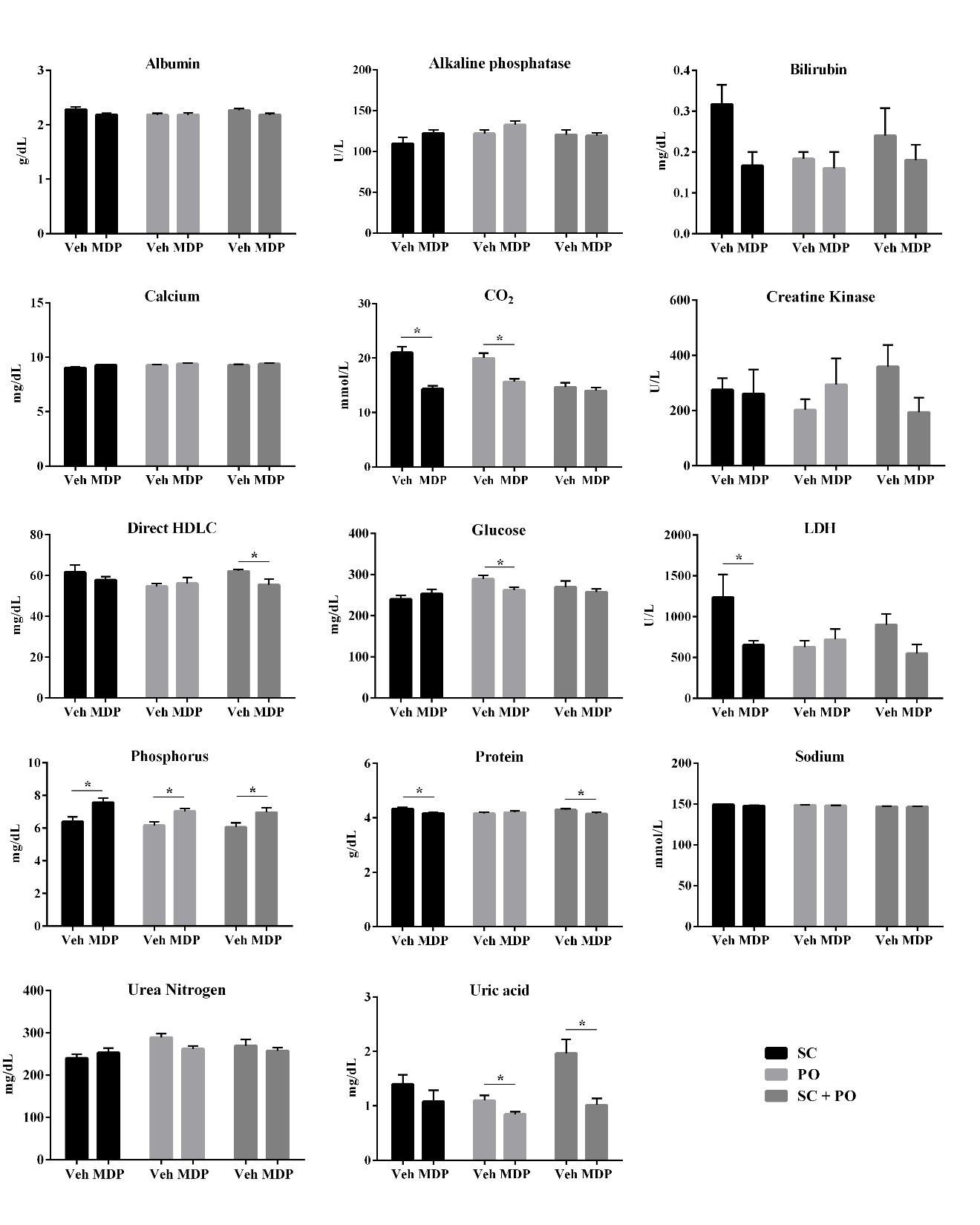
**

Supplement: S4 Fig — Modulation of the various markers of injury albumin, alkaline phosphatase, bilirubin, calcium, CO2, creatine kinase, HDLC, glucose, LDH, phosphorus, protein, sodium, urea nitrogen and uric acid was evaluated in the blood after MDP or vehicle administration. *p < 0.05 vs. Veh via same route. Veh, vehicle; SC, subcutaneous; PO, by mouth. (DOCX) [file pone.0160575.s004.docx]

**S5 Fig:**

**
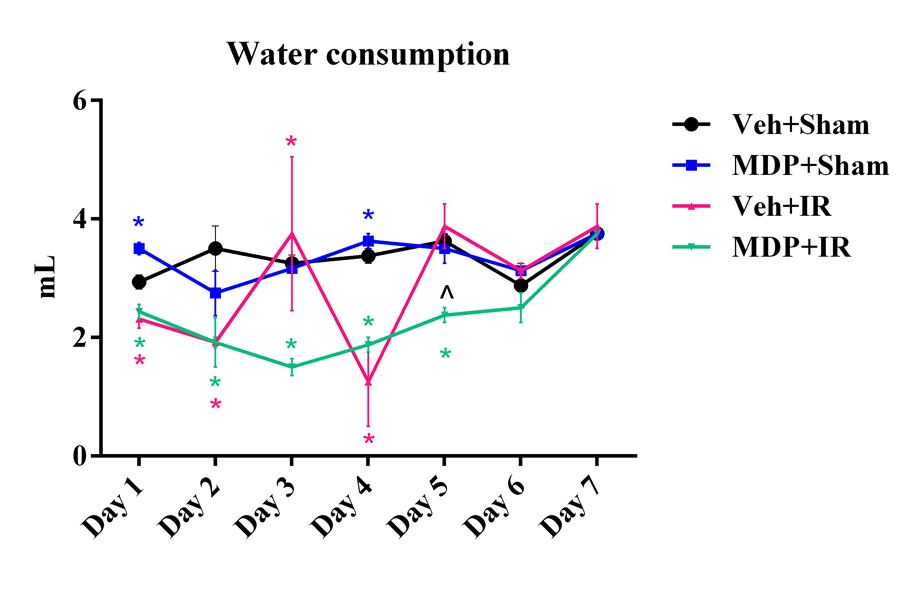
**

Supplement: S5 Fig — Daily water consumption by each animal was measured for the first 10 days post-irradiation. IR exposure dose was 9.5 Gy. *p < 0.05 vs. Veh+Sham; ^p < 0.05 vs. Veh+IR. Abbreviation: Veh, vehicle. (DOCX) [file pone.0160575.s005.docx]

**S6 Fig:**

**
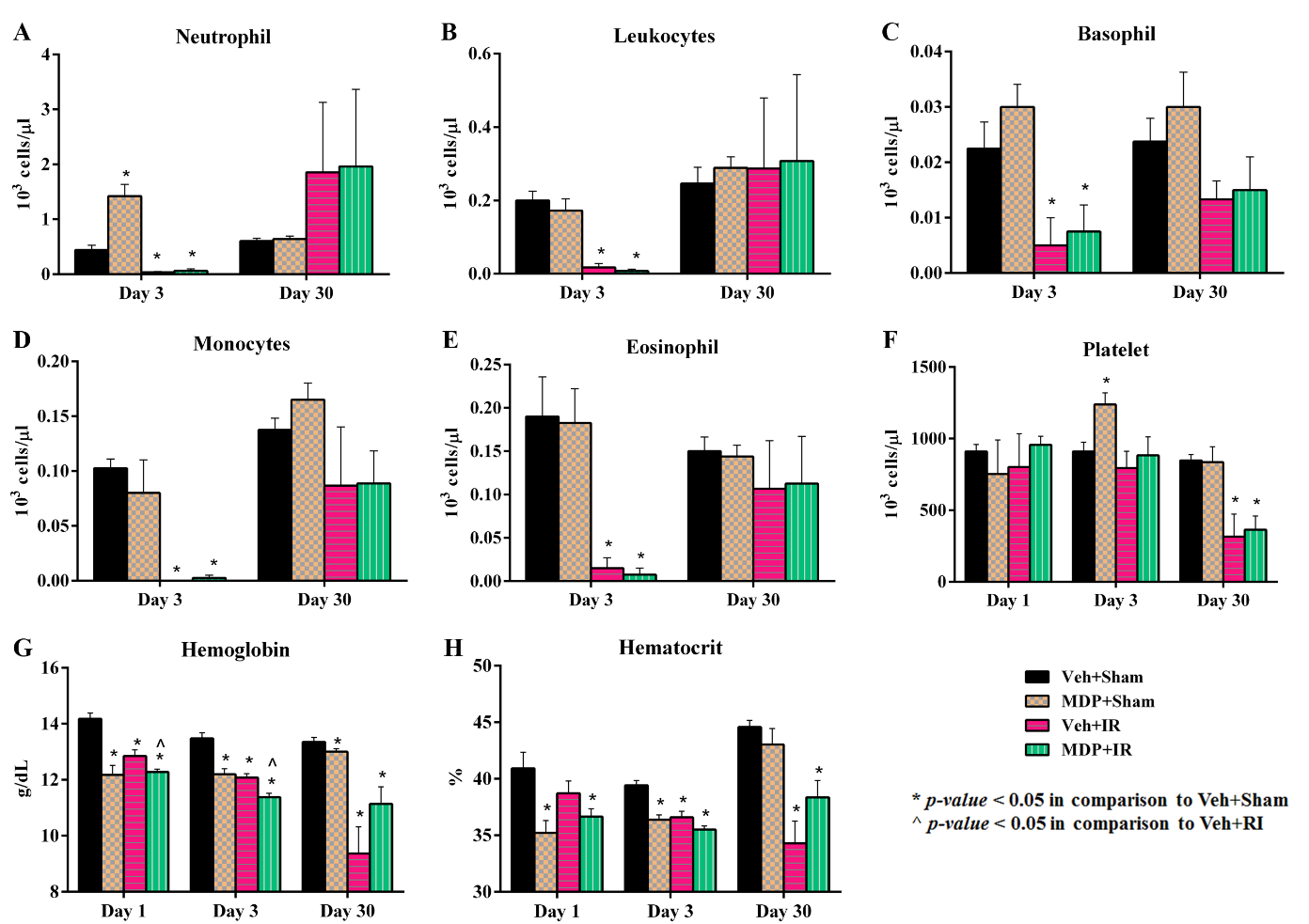
**

Supplement: S6 Fig — Differential cell counting was assayed with blood of the different treatment groups post-irradiation. (A) Neutrophil count, (B) Lymphocyte count, (C) Basophil count, (D) Monocyte count, (E) Eosinophil count, (F) Platelet count, (G) Hemoglobin and (H) Hematocrit levels in blood post-irradiation. IR exposure dose was 9.5 Gy. *p < 0.05 vs. Veh+Sham; ^p < 0.05 vs. Veh+IR. Abbreviation: Veh, vehicle. (DOCX) [file pone.0160575.s006.docx]

**S7 Fig:**


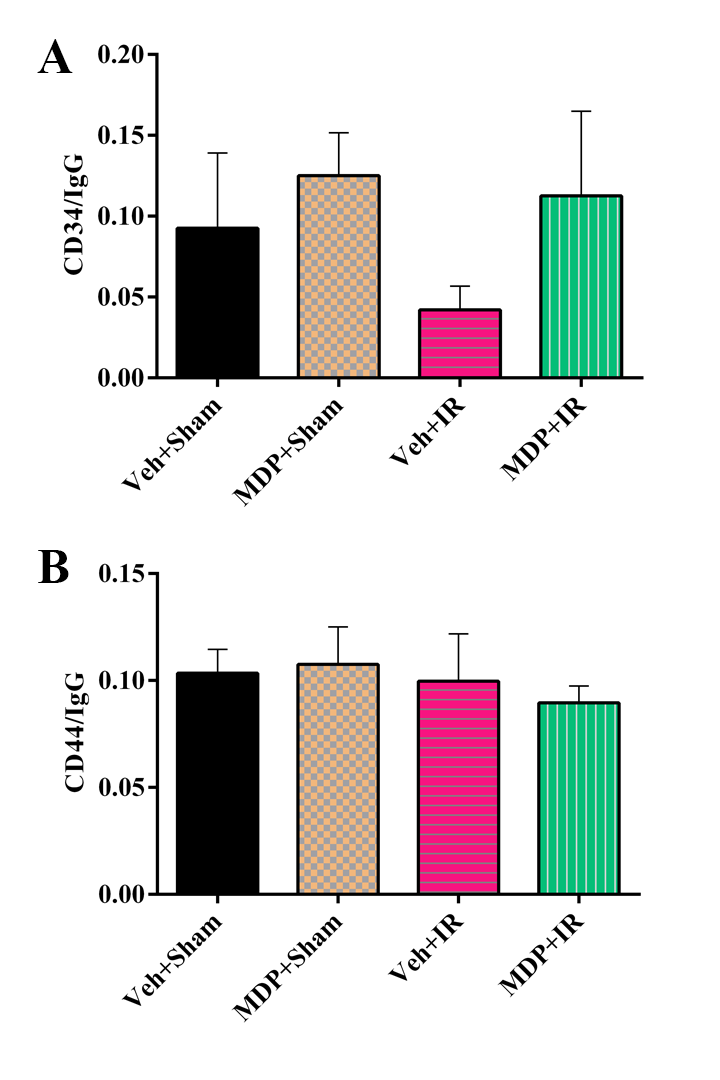

Supplement: S7 Fig — (A) Correlation in the CD34/IgG ratios of the CD34 western blot of bone marrow lysates. (B) Correlation in the CD44/IgG ratios of the CD44 western blot of bone marrow lysates. IR exposure dose was 9.5 Gy. Abbreviation: Veh, vehicle. (DOCX) [file pone.0160575.s007.docx]

**S8 Fig:**


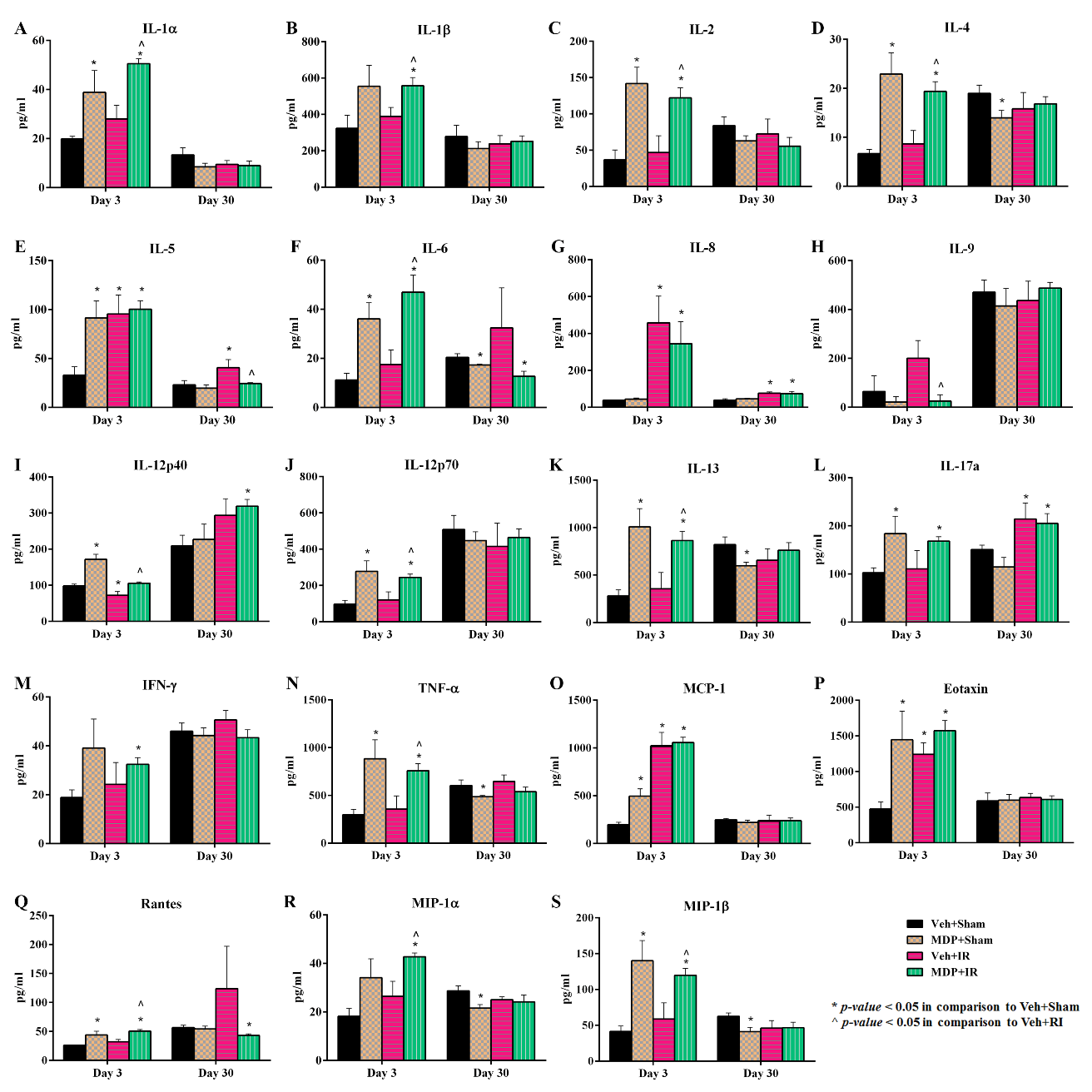

Supplement: S8 Fig — Modulation of cytokine and chemokine expression was assayed in serum on days 3 and 30 post-irradiation by Luminex multiplex array. The panel displays the modulation of (A) IL-1α. (B) IL-1ß. (C) IL-2. (D) IL-4. (E) IL-5. (F) IL-6. (G) IL-8 (KC). (H) IL-9. (I) IL-12p40. (J) IL-12p70. (K) IL-13. (L) IL-17a. (M) IFN-γ. (N) TNF-α. (O) MCP-1. (P) Eotaxin. (Q) Rantes. (R) MIP-1α. (S) MIP-1β. IR exposure dose was 9.5 Gy. *p < 0.05 vs. Veh+Sham; ^p < 0.05 vs. Veh+IR. Abbreviation: Veh, vehicle. (DOCX) [file pone.0160575.s008.docx]
