# Supplementary material for: MDP: A Deinococcus Mn2+-Decapeptide Complex Protects Mice from Ionizing Radiation
Source: PLoS One. 2016 Aug 8;11(8):e0160575. doi: 10.1371/journal.pone.0160575 (PMC4976947; doi:10.1371/journal.pone.0160575)
Supplement: S1 Table — Clinical scoring for Acute Radiation Syndrome (ARS) was done from day 8 to day 30. The table summarizes the clinical scoring criteria and specific score assigned for various categories evaluated. Scores from each category were added together to obtain an overall clinical score for each animal. Animals with an overall clinical score ≥ 12 were considered moribund and humanely euthanized immediately. (DOCX) [file pone.0160575.s009.docx]

**S1 Table: Clinical scoring criteria.** The table summarizes the clinical scoring criteria used during the *in vivo* study.

| **Rodent Intervention Score Sheet** | | |
| --- | --- | --- |
| **PARAMETER** | **DESCRIPTION** | **SCORE** |
| **Appearance** | Normal (smooth coat, clear eyes/nose) | **0** |
|  | Hunched and/or fluffed | **1** |
|  | Ocular discharge and/or edema | **3** |
|  | Emaciated, dehydrated, or soft stools (fecal matter around anus) | **5** |
|  | Bloody diarrhea | **9** |
|  | Blue mucus membranes/skin (cyanosis) | **12** |
| **Respiratory**  **rate** | Normal breathing | **0** |
|  | Increased (double) breathing rate, rapid or shallow | **6** |
|  | Abdominal breathing (gasping +/- open mouth breathing) | **12** |
| **General**  **behavior** | Normal | **0** |
|  | Stretching of hind limbs with abdominal motion (writhe) or grimace | **1** |
|  | Decreased mobility | **2** |
|  | Ataxia, wobbly, appearing weak | **6** |
|  | Unable to stand | **12** |
| **Provoked behavior** | Normal (moves when cage is disturbed or runs from hand) | **0** |
|  | Subdued or weak, responds to stimulation (moves away briskly) | **1** |
|  | Subdued even to stimulation (moves away slowly). | **3** |
|  | Unresponsive to gentle prodding | **6** |
|  | Does not right when placed gently on side within 5 seconds, or no response when the paws are pinched | **12** |
| **Weight loss**  (in comparison to the weight on day (-1)) | <20% | **0** |
|  | 20-25% | **3** |
|  | 26-30% | **6** |
|  | 31-34% | **9** |
|  | >35% | **12** |
|  |  |  |
| **< 6 - Normal** | | |
| **6-11 - Morbid, some pain/distress, monitor at least three times a day.** | | |
| **≥ 12 - Moribund. Euthanize immediately.** | | |
